# Modelling the potential clinical benefit of mobile stroke units in England

**DOI:** 10.1186/s12873-025-01242-8

**Published:** 2025-07-01

**Authors:** Anna Laws, Michael Allen, Jason Scott, Lisa Moseley, Kerry Pearn, Gary A. Ford, Chris Price, Phil White, Graham McClelland, Lisa Shaw, Daniel Phillips, Dave Wilson, Peter McMeekin, Martin James

**Affiliations:** 1 University of Exeter Medical School, Exeter, UK; 2 NIHR South West Peninsula Applied Research Collaboration (ARC), Exeter, UK; 3 Northumbria University, Newcastle-upon-Tyne, UK; 4 University of Oxford, Oxford, UK; 5 University of Newcastle, Newcastle-upon-Tyne, UK; 6 East of England Ambulance Service NHS Trust, Royston, UK; 7 North East Ambulance Service NHS Foundation Trust, Newcastle-upon-Tyne, UK

**Keywords:** Stroke, Mobile stroke unit, Thrombolysis, Thrombectomy

## Abstract

**Background:**

Intravenous thrombolysis (IVT) and mechanical thrombectomy (MT) are well-established emergency reperfusion treatments for stroke caused by clots. Both reduce disability but effectiveness is highly time-dependent, declining in the first few hours after stroke onset. Mobile stroke units (MSUs) have been proposed as a way of improving outcomes after stroke. MSUs enable on-scene brain imaging and delivery of IVT, and can allow for better choice of destination hospital. The primary objective of the study was to model the likely effect of MSUs on clinical outcomes (the ability to live independently, modified Rankin Scale 0–2) across all of England assuming no resource restrictions during deployment.

**Methods:**

We used modelling of times to treatment and outcomes. Modelling was performed for Lower Super Output Areas (LSOAs) in England. Admission numbers were based on Hospital Episode Statistics and travel times estimated from data from Open Street Map. Outcomes were predicted based on times to IVT and MT; we report outcomes as utility or the proportion of patients able to live independently at 3-6 months after stroke. We assumed MSUs and stroke units all had the same propensity to use IVT.

**Results:**

For every 100 patients suitable for IVT or MT, there will likely be 1–3 more people who can live independently following MSU care. The benefit comes from both earlier IVT and the direct transfer of patients likely to benefit from MT to their closest MT-centre by avoiding inter-hospital transfers that would be used in usual care. If, as is likely, about 1 in 5 stroke patients are suitable candidates for IVT or MT, an MSU would need to attend approximately 250 stroke patients for every one extra independent-living outcome. If about half of the patients to whom an MSU is dispatched are actual strokes (the others being stroke mimics), an MSU would need to attend approximately 500 patients for every one extra independent-living outcome. Some areas, furthest from where MSUs are based, will receive no benefit from MSU care, whereas other areas may have up to 4 additional independent-living outcomes for every 100 patients suitable for IVT or MT. Quick MSU dispatch and fast on-scene treatment are crucial to achieving the benefit of MSUs, otherwise use of MSUs may have no overall benefit, or worse outcomes, than usual care. The above benefits do not include any other possible benefits unrelated to earlier IVT or MT.

**Conclusions:**

This study suggests that the overall benefit of MSU care if deployed across all of England is likely to be modest. Selective use of MSUs in specific areas is likely to be more effective than widespread implementation. Rapid dispatch, fast on-scene treatment of patients, and careful selection of which patients to dispatch the MSU to (by location and confidence in that person being a confirmed stroke patient), are all critical for maximising benefits from MSU care. MSUs should not be seen as an alternative to optimising day-to-day emergency stroke systems.

**Supplementary information:**

The online version contains supplementary material available at 10.1186/s12873-025-01242-8.

## Introduction

Stroke remains one of the top three global causes of death and disability [1]. Despite reductions in age-standardised rates of stroke, ageing populations are driving an increase in the absolute number of strokes [1]. Across Europe, in 2017, stroke was found to cost healthcare systems €27 billion, or 1.7% of health expenditure [2].

Intravenous thrombolysis (IVT) and mechanical thrombectomy (MT) are two well-established treatments for reduction or removal of clots causing ischaemic stroke, reducing disability caused by stroke, but both lose effectiveness in the hours following a stroke [3, 4]. IVT is suitable for both non-large vessel occlusions (nLVO) and large vessel occlusions (LVO). MT is suitable only for LVO, but has superior efficacy compared to IVT.

As a way of improving time from onset to IVT, mobile stroke units (MSUs) were first proposed in 2003 by Fassbender et al. [5]. An MSU is a bespoke ambulance that contains the required technology and specialist skills for diagnosing ischaemic stroke and making an IVT treatment decision, i.e. a computed tomography (CT) scanner and an in-person or telemedicine review by a stroke specialist [6]. MSUs were first trialled in Homberg, Germany [7] and have been introduced based on MT-capable centres (comprehensive stroke centres, CSCs) in other health systems. No health system has yet deployed MSUs at a regional or country-wide level.

Fatima et al. [8] have published a meta-analysis of MSU trials. They reviewed evidence from a total of 21,297 patients from 11 publications (seven randomized controlled trials and four non-randomized controlled trials including prospective cohort studies). Mean time to IVT was reduced by 12 min on average, from 75 to 63 min. In a pooled analysis they found the odds of a good outcome (modified Rankin Scale, mRS, 0–2 at day 7) were improved 1.46 times by use of an MSU.

In a separate meta-analysis, Chen *et al.* [9] reviewed evidence from a total of 22,766 patients from 16 publications. In total 7,682 (33.8%) were treated in the MSU and 15,084 (66.2%) with usual care. They found higher use of IVT in the MSU group (37.3% vs. 27.7%), and an improvement in the proportion of patients with mRS 0–2 at 90 days (66.2% vs 58.8%). The pooled analysis of time metrics indicated a mean reduction of 33 min in time to IVT between MSU and usual care.

Turc *et al.* [10] conducted a further meta-analysis that reviewed evidence from 14 studies. They found that MSUs increased the odds of a better outcome by 1.64 (adjusted odds ratio), with a median reduction in time to IVT of 31 min and an increase in odds of receiving IVT of 1.83 (unadjusted odds ratio). The odds of receiving IVT in the first 60 min after stroke (the golden hour) were increased by 7.71 (unadjusted odds ratio).

There is less known about how MSUs affect use, and speed, of MT, where the main advantage of use of a MSU with CT-A would be direct transfer of the patient to a MT-capable centre (avoiding inter-hospital transfers with their associated delays). A review of available evidence suggests there is an evidence gap in MSUs and MT, as in many trials the MSU were dispatched from CSCs, into populations which would have been directly admitted under usual care [11]. In Berlin, Ebenger *et al.* [12] found that the MT rate and speed were essentially unchanged (13. 8% vs 14. 2%, and the median dispatch to MT 137 min vs 125 min, for MSU and usual care). In the BEST Study [13] median time from 911 alert to MT was 141 and 132 min for MSU and usual care. The BEST-MSU substudy [14] was a subset of the BEST study, for IVT-eligible stroke patients with LVO MT rates were 79% and 86% for MSU and usual care, with median alert-to-puncture times of 142 min and 132 min. In general, in that study, for patients with LVO stroke eligible for IVT, though MT was delivered a little later, MSU management was associated with better clinical outcomes compared to standard management, with an adjusted odds ratio of having mRS 0–1 of 2.60. Helwig *et al.* [15] showed the potential of MSUs for improved triage of patients to the correct type of hospital (providing MT or not). Compared to the Los Angeles Motor Scale (LAMS), MSUs had a triage accuracy of 100% compared to about 70% for LAMS. MSUs equipped with CT Angiogram (CT-A) were also found to significantly improve MT workflows at the receiving hospital, reducing door-to-puncture times from 94 to 43 min [16].

MSU trials have generally been in metropolitan areas, where the MSU can respond rapidly to suspected stroke and travel times are relatively short. In order to predict the benefit of MSUs for patients with suspected LVO across a broader geographic area, Holodinsky et al. [17] used modelling to predict the benefit, from IVT and MT, of using MSUs to cover larger distances (up to 4.5 h travel for the MSU, to include all people who might receive IVT). They found that the effect of MSUs was likely to be minimal in the regions close to the stroke centre that was the MSU base. They predicted no more than 1 percentage point increase in the proportion of patients with an outcome of mRS 0–2, but this could rise to about a 2 percentage point increase in a halo further away from the stroke centre where the MSU made the difference between receiving IVT or not.

Our work aimed to model the effect of MSUs on the likely times to both IVT and MT (including modelling inter-hospital transfers when required) and to estimate outcomes both as the proportion with mRS 0–2 at 3-6 months and their corresponding health utility. We modelled these effects across all of England (population 58 million in 2023) performing a detailed geographic analysis at the Lower Super Output Areas (LSOA) level. We performed a comprehensive analysis of the effect of changing process times for usual care and MSU care. We also investigated how the number of MSU base locations affects time to IVT/MT and resulting patient outcomes.

## Methods

### Method overview

Strokes are defined as either an nLVO or LVO, with their treatment options being IVT (for an nLVO) or IVT followed by MT (for an LVO). This work compares the outcomes of patients under two treatment delivery models: i) *usual care*, in which the patient is taken to their nearest emergency hospital for IVT, and for patients with with onwards transfer to a MT-capable centre for patients considered likely to eligible for MT (which involves additional travel time and other transfer-related delays); ii) *MSU care*, in which an MSU attends the patient on-scene to provide IVT, with patients with an LVO (shown on an on-scene CT angiogram) being taken directly to their most local MT-capable centre. The scope of the modelling includes the stroke patients’ pathway from stroke onset to treatment with IVT and, where appropriate, MT, with this time to treatment being translated to the patient outcome, which is dependent also on the stroke type (nLVO or LVO) and treatment received. We explore sensitivities around pathway process durations using scenario analysis, the variation due to the stroke onset location using geographic analysis, and the impact of the number of MSU base locations on patient outcomes. It was assumed throughout that there were no resource restrictions upon the use of MSUs, with complete implementation across all areas, and that all eligible patients with an LVO would receive MT. The maximum time from stroke onset to receiving treatment was 4.5 h for IVT, and 8 h for MT - configurations of MT base locations may be chosen such that for some patients treatment would not be possible due to process times exceeding these treatment time thresholds.

Our modelling compares outcomes for those patients receiving IVT or MT. We do not make any assumption about the proportion of all patients receiving those treatments.

### Lower Super Output Areas (LSOA)

We performed our analysis across England using LSOA as our geographic footprint [18]. LSOAs are small areas in England, with roughly 1,500 people in each area. There are 32,843 LSOAs in England.

### Stroke admission data

Stroke admission numbers per LSOA were taken from Hospital Episode Statistics (HES) 2017–2019 (using ICD-10 codes of I61, I63 and I64). Across those three years, in total there were 242,874 recorded stroke admissions coming from an England LSOA going to one of the 101 acute stroke units in England (an average of 2.47 admissions per LSOA per year). The stroke patient population were divided into stroke type cohorts, based on vessel occlusion type (LVO and nLVO). Using analysis from reperfusion treatment clinical trials [3, 4, 19, 20], we classify each patient’s vessel occlusion type based on their National Institutes of Health Stroke Scale (NIHSS) on arrival (NIHSS 0–10 as nLVO; NIHSS 11 + as LVO), as NIHSS has been shown to have higher accuracy in separating nLVO and LVO than other stroke scales (Area Under the Receiver Operating Characteristic Curve = 0.86 [21]). Applying this classification to the stroke admission data in England and Wales (Sentinel Stroke National Audit Programme), this provides an estimate of 30% LVO and 70% nLVO in the potentially treatable population. This proportion split was applied to each of the LSOAs in the study (to divide the total stroke admissions by LSOA as provided by HES into these two patient stroke type cohorts). These derived values were also cross-checked against stroke types identified in studies on pre-hospital selection of patients with suspected LVO [22], where LVO made up 38% of the population where a pre-hospital diagnostic (RACE score) was applied. Population density was taken from the Office of National Statistics 2011 census.

### Pathway processes modelling

All code used for usual care and MSU care pathway modelling is available [23].

**Fig. 1:**
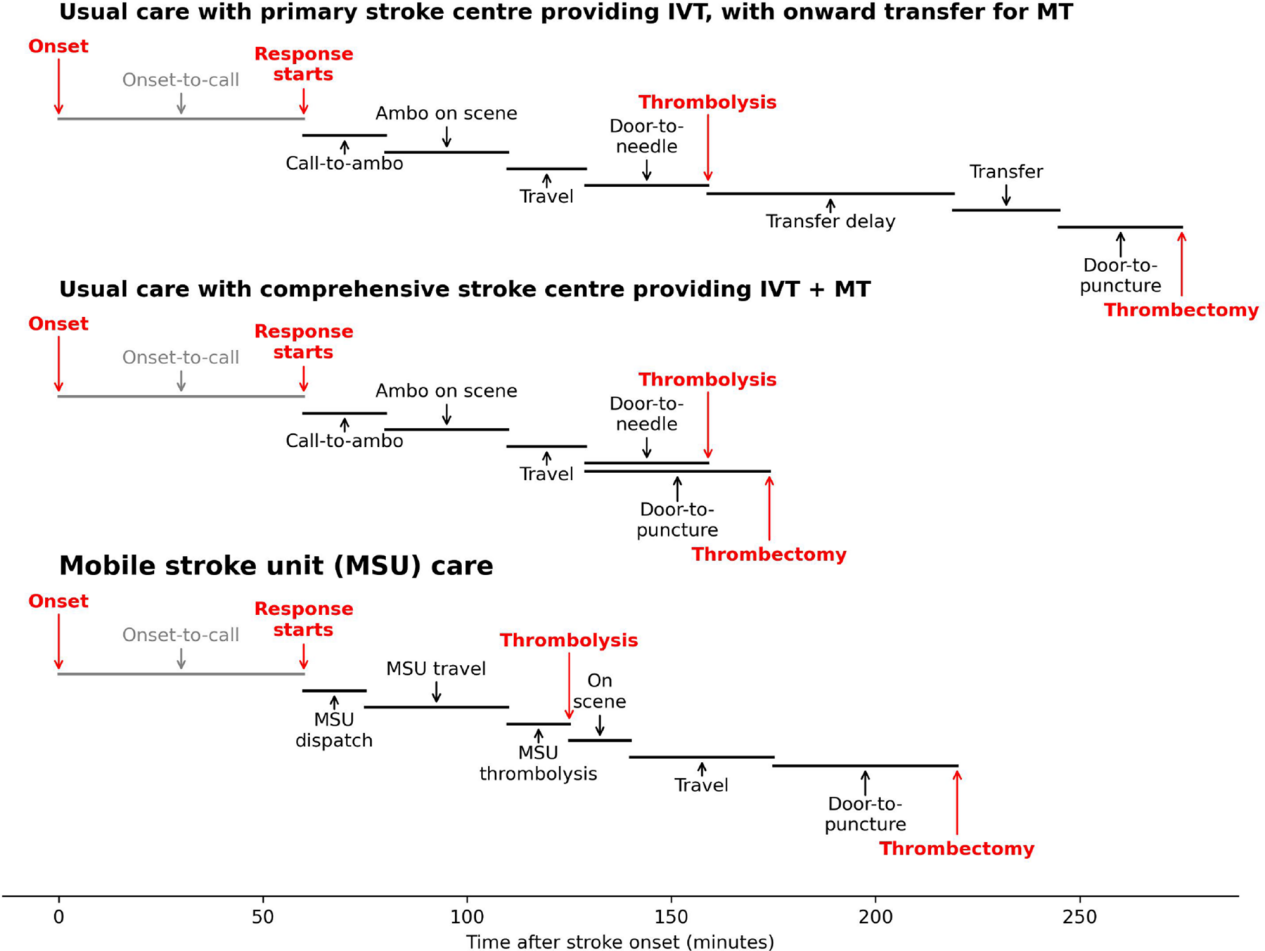
An illustrative timeline showing processes included in the three pathways that are modelled for provision of IVT and MT. Top: Usual care pathway for patients with a PSC closest to their home LSOA, with the PSC providing IVT, followed by the LVO patients having a transfer to the nearest CSC for MT. Middle: Usual care pathway for patients with a CSC closest to their home LSOA, with the CSC providing both IVT and MT. Bottom: MSU care pathway, with IVT provided on-scene by the MSU, followed by the MSU transferring the LVO patients to the nearest CSC for MT. Process times other than travel times are common for all patients (defined by the scenario). Travel times depend on locations of patient and hospitals, with results calculated for all LSOAs in England. n.b. “ambo” is short for “ambulance”

Figure 1 describes the processes included in the three pathways that are modelled: two pathways for usual care depending on which type of stroke centre is nearest to the LSOA, and one pathway for MSU care. *Usual care* is provided by the patient attending their nearest stroke centre, which is either i) a *primary stroke centre*, PSC, providing IVT only, with onward transfer for LVO patients requiring MT to the *comprehensive stroke centre* that is nearest to the PSC, or ii) a *comprehensive stroke centre*, CSC, providing both IVT and MT. The *MSU care* pathway involves the MSU providing on-scene IVT, followed by the MSU transferring the LVO patients to the closest CSC for MT. The onwards transfer of nLVO patients is not included in the scope of this modelling.

Based on input from three co-production workshops [24] involving representation from stroke consultants, ambulance staff and patients and public (4–6 from each group at each of three workshops), we designed the model to explore large numbers of possible parameter values (see *scenario analysis* section for more detail). This reflected the uncertainty of how the MSUs would operate and perform if implemented.

Unless specified otherwise, it was assumed that MSUs are located at CSCs. We also assume MSUs would be equipped with CT angiography (CT-A) and can identify LVO patients who will benefit from direct transfer to a CSC for MT.

Geographic analysis was undertaken at LSOA level. Travel times from each of the 32,843 LSOAs in England to all hospitals (PSC and CSCs), and travel times between hospitals have been estimated using Open Street Map data, with results calibrated against Google Maps. Travel times have been made available [25].

For each LSOA, times to IVT and MT are calculated by summing all the non-travel process times (common for all patients and defined by the scenario) and adding required travel times (bespoke for each LSOA location, and for each inter-hospital transfer). The next section will describe how patient outcomes are calculated based on these LSOA-specific times to IVT and MT for usual care or MSU care. All calculations are performed in Python/NumPy.

### Outcome modelling

Outcome modelling is based on published meta-analysis of clinical trials which have evaluated the decline of effectiveness of IVT and MT over time [3, 4]. We have applied those models to the expected real world treatment population (based on the national stroke audit of England and Wales, SSNAP). We report the expected benefit in the treated population for nLVO and LVO separately, and also using a 70:30 mix of nLVO and LVO derived from the patients currently arriving at hospital within 4 h of stroke onset - as this is likely to be similar to the population where a MSU is dispatched and IVT treatment is given on-scene.

Detailed methods and code used for modelling these outcomes are available [26], with methods described in the appendix and as an online book [27]. The outcome model is available as a PyPI package for Python [28].

We used modified Rankin Scale (mRS) at 3–6 months as a measure of outcome. mRS is the most commonly used instrument to describe post-stroke functional outcome [29], describing independence of living from a scale of 0 (no disability) through to 5 (severe disability requiring constant nursing attention), with death assigned an mRS of 6. A commonly used surrogate for independent living is mRS 0–2. Health utility values for each mRS level were taken from Wang *et al.* [30]. The mean mRS score, mean utility and proportion of patients with mRS 0–2 in a given mRS distribution can be compared between scenarios.

We calculated the patients’ mRS outcome distribution based on time to treatment for three patient-treatment cohorts: nLVO treated with IVT; LVO treated with IVT alone; and LVO treated with IVT and MT. For each patient-treatment cohort we calculated an mRS distribution for treatment at any given time by interpolating between the mRS distribution for treatment given at *t = 0* (time of stroke onset) and the mRS distribution for treatment given at *t=No Effect* (time of no effect of treatment), assuming that log odds fall linearly over time [3, 4]. Further details on how these *t = 0* and *t=No Effect* mRS distributions were derived are given in the supplementary material.

The time to no effect was 6.3 h for IVT [3] and 8.0 h for MT [4]. Our model did not include selection of patients who may still benefit from treatment beyond these durations through the use of perfusion scanning. This number is small for IVT, but is more substantial for MT – approximately 2500 per annum in England.

### Scenario analysis

Scenario analysis was undertaken to investigate how changing assumed model parameters (the process durations, in minutes) affect outcomes across all LSOAs. The parameter values were varied according to the following, with all combinations modelled:

- All patients:
  - Stroke onset to call: 0, 60, 120, 180

- Usual care:
  - Call to ambulance arrival: 15, 30, 45
  - Ambulance on-scene: 20, 30, 45
  - Hospital arrival to IVT (door-to-needle): 30, 45
  - Transfer-related net MT delay (excluding travel time): 30, 60, 90
  - Hospital arrival to MT (door-to-puncture): 60, 90, 120

- Mobile stroke units:
  - Call to MSU dispatch: 0, 15, 30, 45
  - MSU arrival to IVT: 15, 30, 45
  - MSU on-scene post-IVT: 5, 15
  - MSU hospital arrival to MT (door-to-puncture): 30, 60, 90

At the CSC in the usual care pathway, the *hospital arrival to MT* time has been shown to be shorter for patients transferred from a PSC than for patients who are directly admitted [31]. This is due to some of the necessary processes (such as information gathering, imaging and IVT) already being completed at the PSC. However, these patients will experience a delay in receiving MT (in addition to their inter-hospital travel time) due to waiting for transfer from the PSC. These two time durations (additional time waiting for transfer and shorter *hospital arrival to MT*) are represented in the model as a single parameter: the *transfer-related net MT delay* parameter. For example, if the time spent in the PSC is 90 min (known as the *door-in-door-out* time), but the *hospital arrival to MT* is reduced by 30≤minutes, this is represented by setting *transfer-related net MT delay* to 60≤minutes in the model and leaving the *hospital arrival to MT* unchanged.

### Geographic analysis

To study geographic variation in benefit of MSU care, a single set of parameters was chosen that reflected a reasonable base case for performance of usual care and MSU care. Process times (minutes) are shown in the following list, with ambulance and MSU travel times and inter-hospital travel times dependent on patient location (LSOA). In this base case scenario MSUs are based at CSCs only.

- All patients:
  - Stroke onset to call: 60

- Usual care:
  - Call to ambulance arrival: 20
  - Ambulance on-scene: 30
  - Hospital arrival to IVT: 45
  - Transfer-related net MT delay (excluding travel time): 60
  - Hospital arrival to MT: 90

- Mobile stroke units:
  - Call to MSU dispatch: 15
  - MSU arrival to IVT: 30
  - MSU on-scene post-IVT: 5
  - MSU hospital arrival to MT: 60

### Varying number of MSU base locations

In order to study the effect of changing the number of MSU base locations, a greedy algorithm was used. In this method, 100 MSU base locations are sequentially added, with each additional one chosen from the 101 current acute stroke units (of any type, CSC or not), in the order of maximum improvement in outcomes (utility). The utility gain is calculated for those patients treated by an MSU rather than usual care; the algorithm is therefore looking at the effect of changing the number of MSU base locations, rather than the number of MSU vehicles. The algorithm was also run with MSU base locations chosen from only the 23 current CSCs.

## Results

### Scenario analysis

**Fig. 2:**
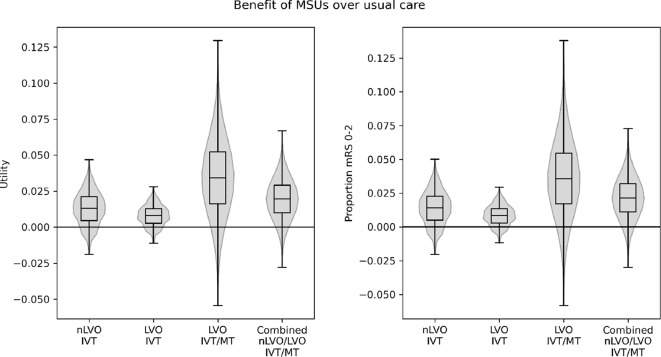
Benefit of MSU care over usual care across all scenarios (exploring the effect of changing process durations), in the treated population, measured by utility (left) or the proportion of patients with an outcome of mRS 0–2 (right), separating the changes for patients with nLVO and LVO. Results for LVO patients show the effect of MSU care on the benefit derived from IVT alone, or by IVT/MT in combination. Box plots show range, interquartile range, and median across all scenarios. The combined nLVO/LVO benefit assumed 70% nLVO and 30% LVO in the treated population, with LVOs receiving IVT/MT in combination. Overlaid on the box plots are violin plots showing the distribution of results across all scenarios. A positive value indicates MSU care provides an advantage over usual care, and a negative value indicates MSU care is disadvantageous compared with usual care. Results are the average effect across all LSOAs in England

Figure 2 shows the range of benefit, measured by utility or the proportion of patients with an outcome of mRS 0–2, across all of the scenarios to explore the effect of changing the process durations, with changes shown both separately for nLVO and LVO and for a combination assuming 70% nLVO and 30% LVO in the treated population. For the combined nLVO/LVO population, compared with usual care, MSU care has a net improvement in: the proportion of patients with an outcome of mRS 0–2 and a net improvement in utility for 92% of all scenarios; the proportion of patients with an outcome of mRS 0–2 of at least 0.01, 0.02, 0.03, or 0.04 in 77%, 53%, 29%, and 11% of all scenarios respectively; and utility of at least 0.01, 0.02, 0.03, or 0.04 in 75%, 49%, 23%, and 7% of all scenarios respectively. Across the majority of scenarios the benefit of MSU care over usual care was typically (interquartile range across scenarios) an improvement of the proportion of patients mRS 0–2 of 0.005 to 0.023 for nLVO, 0.017 to 0.055 for LVO treated with both IVT and MT, and 0.011 to 0.032 for the combined population. This translated to an improvement of 0.005 to 0.021 in utility for nLVO, 0.016 to 0.052 for LVO treated with both IVT and MT, and 0.010 to 0.029 for the combined population. The benefit to patients with LVO was derived mostly by increasing the benefit from MT due to faster treatment times after direct transport to a CSC.

**Fig. 3:**
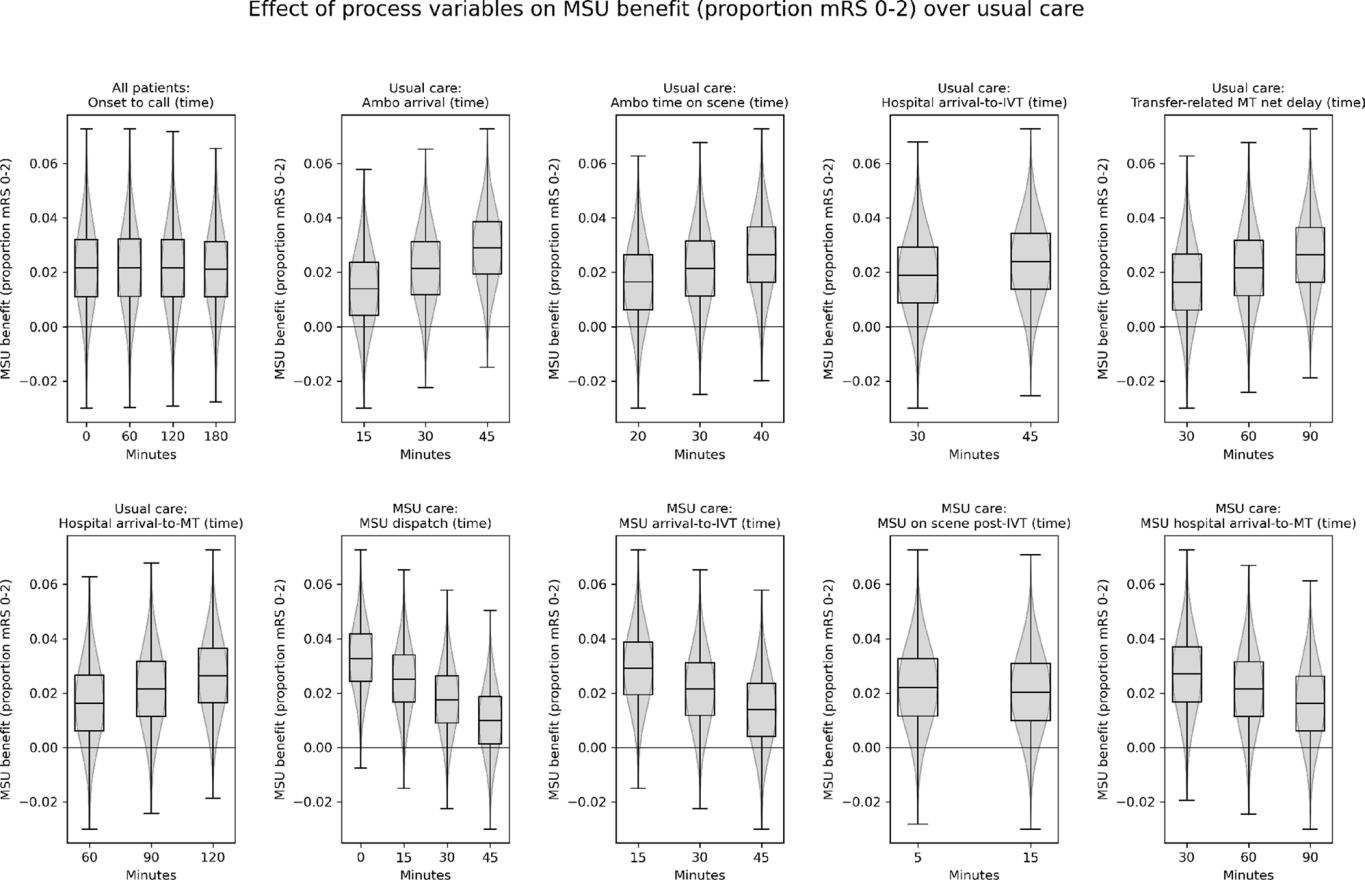
The effect of changing modelled process durations on the predicted benefit of MSU care over usual care, in the treated population (comprised of 70% nLVO and 30% LVO patients, where nLVO patients receive IVT and LVO patients receive IVT followed by MT), measured by proportion of patients with an outcome of mRS 0–2. For each target parameter, results are averaged across all scenarios with that given parameter value. Box plots show range, interquartile range, and median, across all scenarios. Overlaid over the box plots are violin plots showing the distribution of results across all scenarios. Results are the average effect across all LSOAs in England

**Fig. 4:**
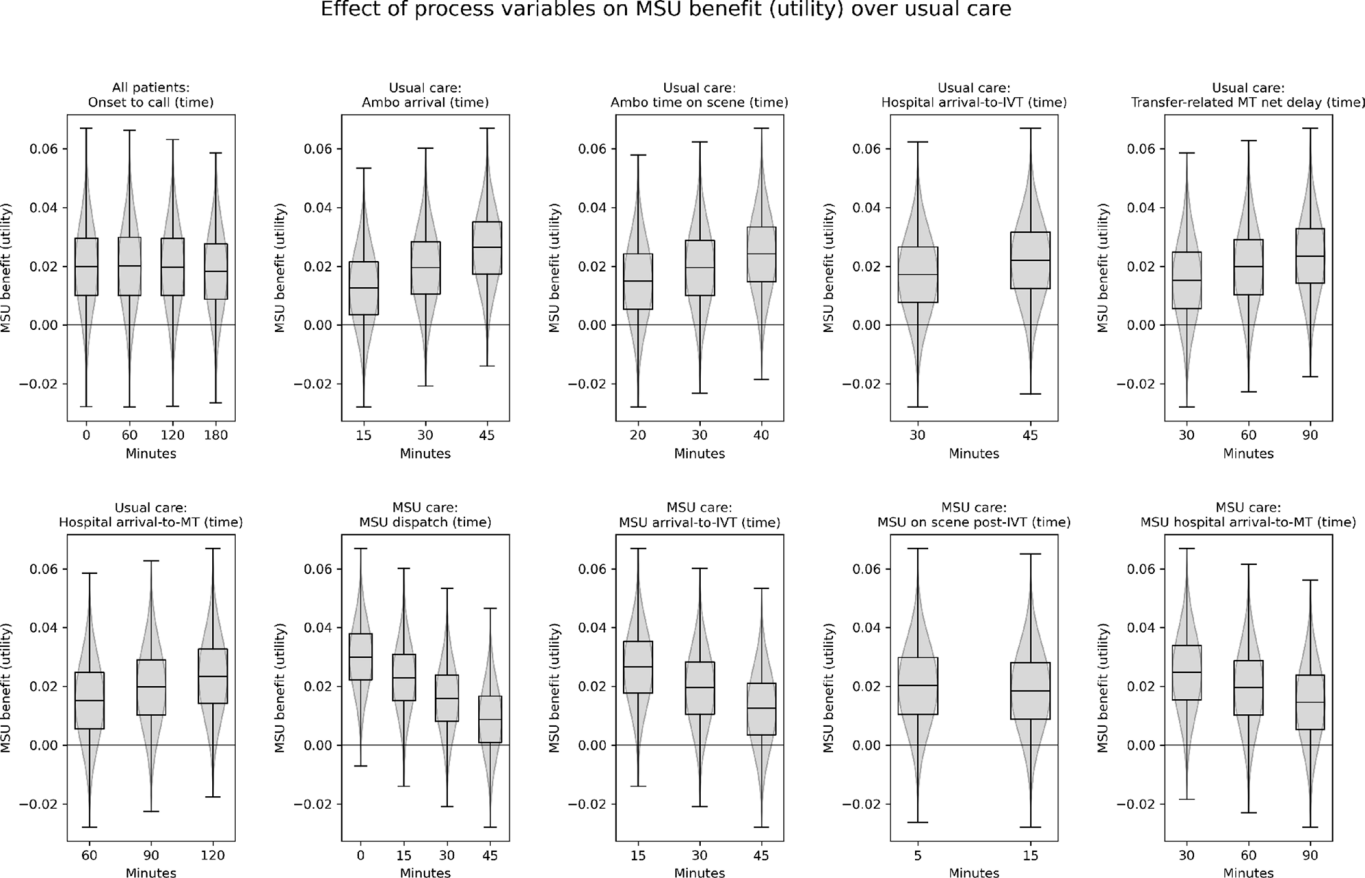
The effect of changing modelled process durations on the predicted benefit of MSU care over usual care, in the treated population (comprised of 70% nLVO and 30% LVO patients, where nLVO patients receive IVT and LVO patients receive IVT followed by MT), measured by utility. For each target parameter, results are averaged across all scenarios with that given parameter value. Box plots show range, interquartile range, and median, across all scenarios. Overlaid over the box plots are violin plots showing the distribution of results across all scenarios. Results are the average effect across all LSOAs in England

Figures 3 and 4 show how changing modelled process durations affects the predicted benefit of MSU care, expressed in proportion of patients with an outcome of mRS 0–2 or utility, respectively. Results show the combined effect on nLVO/LVO, with patients with LVO receiving IVT/MT in combination. Changing time from onset to call only had marginal effect on the benefit of MSU care over usual care. Changing parameters that worsen usual care (such as lengthening ambulance response times, or lengthening arrival to IVT), lead to increased advantages of MSU care over usual care. Likewise, changing parameters that improve MSU care (such as MSU dispatch times, or time to IVT on scene) increased the advantages of MSU care over usual care.

### Geographic variation

For this comparison, the MSUs are based at CSCs only and we assume there is a mix of 70% nLVO and 30% LVO in the treated population in which nLVO patients receive IVT and LVO patients receive IVT followed by MT.

**Fig. 5:**
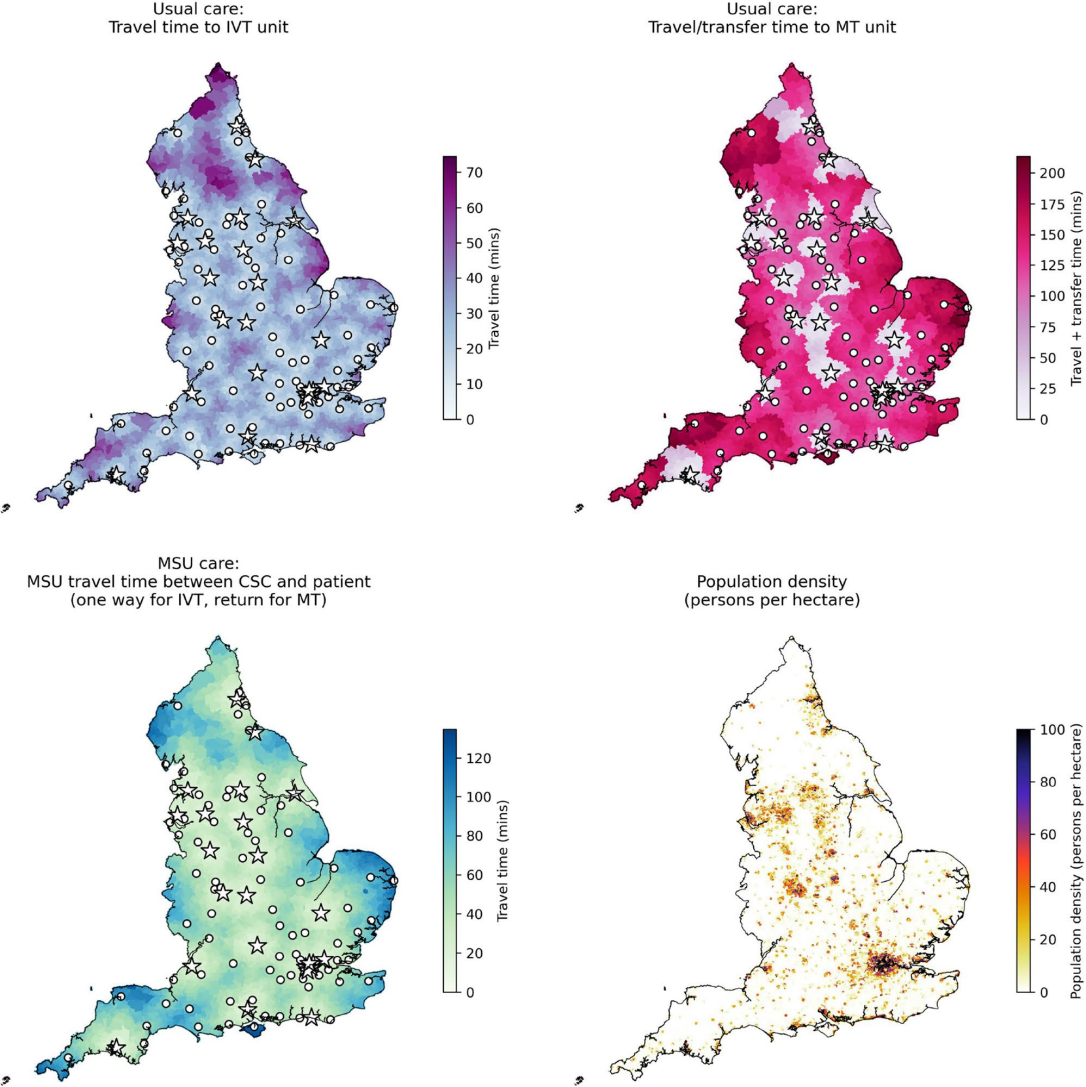
Travel times to treatment under the two treatment delivery models, and population density for each LSOA. *Top left*: With *usual care* the travel times from the patients LSOA to their nearest PSC (providing only IVT). *Top right*: With *usual care* the travel and, where necessary, transfer times from the patients LSOA to a CSC (providing MT). For those patients that first attend a PSC providing only IVT, the times shown include the travel time to the PSC, a net additional delay of 60 min, and the inter-hospital travel time between PSC and CSC. *Bottom left*: With *MSU care* with the MSU base locations at the current 23 CSCs, the travel times for the MSU between the patients LSOA and their nearest CSC (one way for travel time to IVT, return journey for travel time to MT). *Bottom right*: Population density (with scale capped at 100 persons per hectare). Circles show locations of PSCs (providing only IVT). Stars show locations of CSCs (providing both IVT and MT), and being the base locations of MSUs

Figure 5 shows travel and transfer times for usual care and for MSU care. Under usual care, when a patient first attends a PSC (providing only IVT), the travel/transfer time to MT include the travel time to the nearest PSC, and a net additional 60 min delay (taking into account the *door-in-door-out* time at the first admitting hospital and a reduction in time to MT at the CSC for transferred patients). Under usual care, there is significant variation in travel times from patient LSOA to the first attended centre (PSC or CSC) for IVT, but there is more substantial variation in times to MT due to some patients (67% of LSOAs and 70% of admissions) requiring a transfer for MT. Under MSU care, as the MSUs are based at CSCs only, travel times for an MSU to deliver IVT can exceed the travel time of a normal ambulance to the closest centre providing IVT, and this extra travel time can lead to delayed time to IVT in those LSOAs furthest away from MSU base locations. However, for patients living in LSOAs that are close to a CSC, but with a PSC even closer, these patients benefit from MT sooner under MSU care than compared to usual care as they avoid the delays relating to attending the PSC first.

**Fig. 6:**
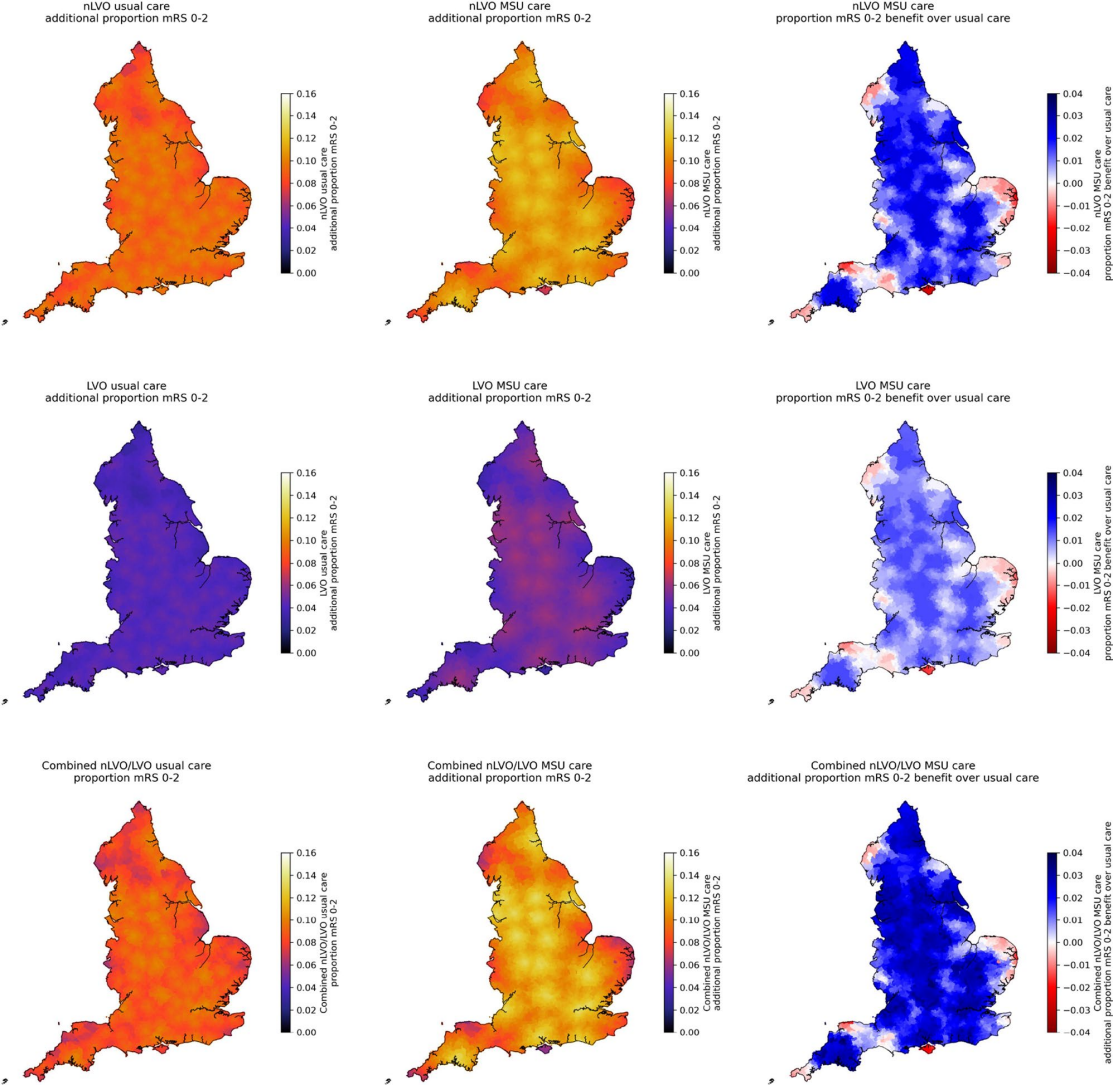
Map of treatment benefits expressed as the proportion of patients with an outcome of mRS 0–2, calculated by LSOA for the treated population. *Top*: Treatment of nLVO; *Middle*: Treatment of LVO, *Bottom*: Combination of treatment (based on 70% nLVO and 30% LVO in the treated population, with LVO receiving IVT/MT in combination). *Left*: Benefit of usual care over no treatment; *Middle*: Benefit of MSU care over no treatment; *Right*: Benefit of MSU care over usual care. Circles show locations of PSCs (providing only IVT). Stars show locations of CSCs (providing both IVT and MT, and are also the base locations of MSUs)

**Fig. 7:**
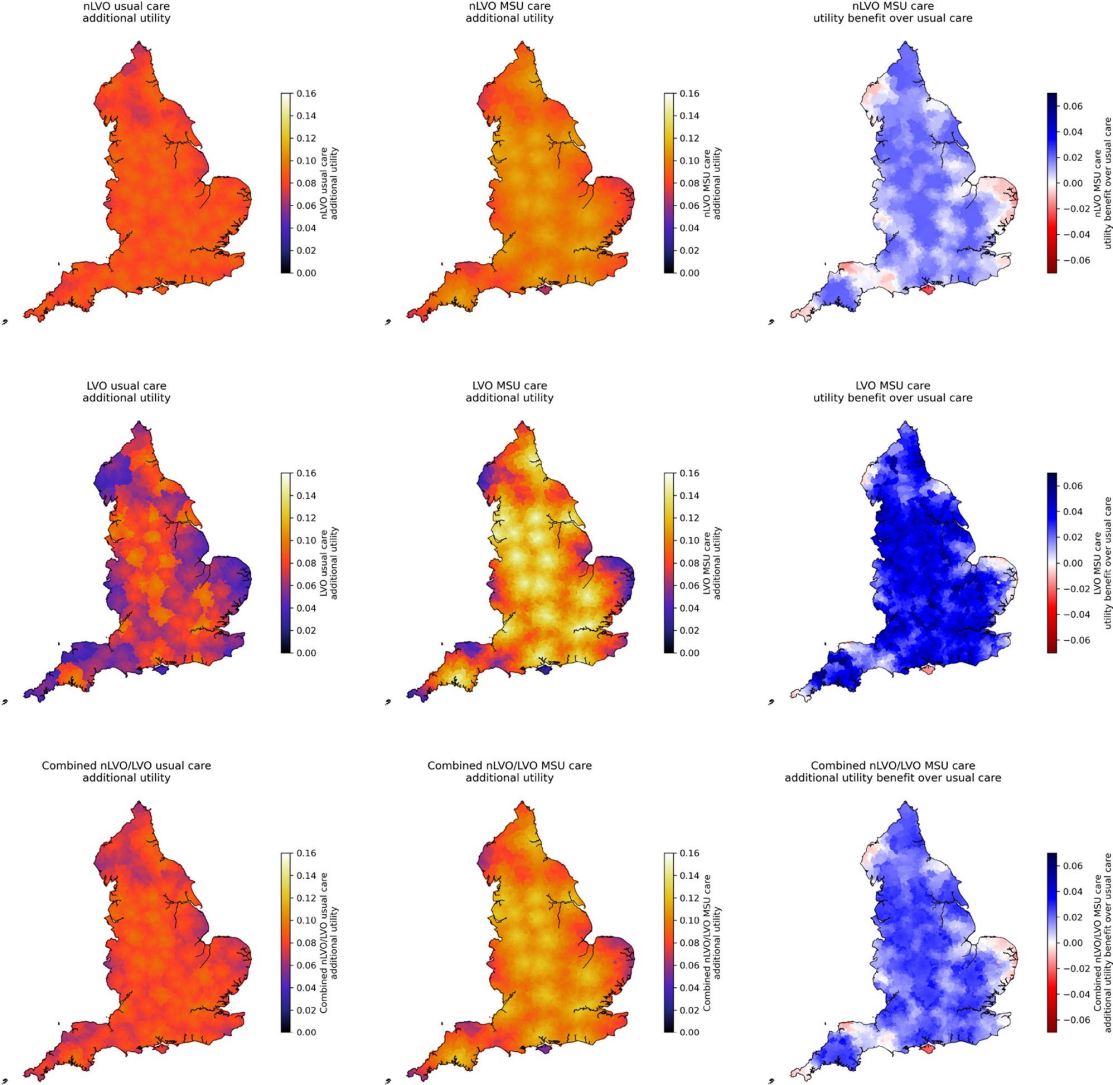
Map of treatment benefits expressed as the utility benefit, calculated by LSOA for the treated population. *Top*: Treatment of nLVO; *Middle*: Treatment of LVO, *Bottom*: Combination of treatment (based on 70% nLVO and 30% LVO in the treated population, with LVO receiving IVT/MT in combination). *Left*: Benefit of usual care over no treatment; *Middle*: Benefit of MSU care over no treatment; *Right*: Benefit of MSU care over usual care. Circles show locations of PSCs (providing only IVT). Stars show locations of CSCs (providing both IVT and MT, and are also the base locations of MSUs)

Figures 6 and 7 show maps (by ischaemic stroke subtype) of geographic variation in benefit (expressed in proportion of patients with an outcome of mRS 0–2 or utility, respectively) between treatment delivery models. Overall, with no reperfusion treatment, there is an average outcome utility of 0.52 across the combined population. With usual care, there is a utility gain of 0.086 in treated patients. MSU care provided an average further utility gain in treated patients of 0.022 over usual care. There is significant variation in the benefit of reperfusion using usual care, with those living closest to CSCs receiving the greatest benefit. This is due to the larger utility gain of reperfusion treatment coming from the treatment of LVO, and with those patients benefiting most from rapid access to MT.

The benefit of MSU care over usual care follows different patterns for nLVO and LVO patients. For nLVO patients, the benefit of MSU care decreases with increased distance from the MSU base location. For LVO patients the benefit of MSU care first decreases with increased distance from the MSU base location, but then benefit increases where use of the MSU avoids inter-hospital transfer for MT, before decreasing again with greater travel times for the MSU. The catchment area of benefit for LVO under MSU care is therefore wider than the catchment area of benefit for nLVO, with the maximum benefit being in a halo a little distance from the MSU base location, where patient transfer for MT is avoided but MSU travel times are still acceptable.

**Fig. 8:**
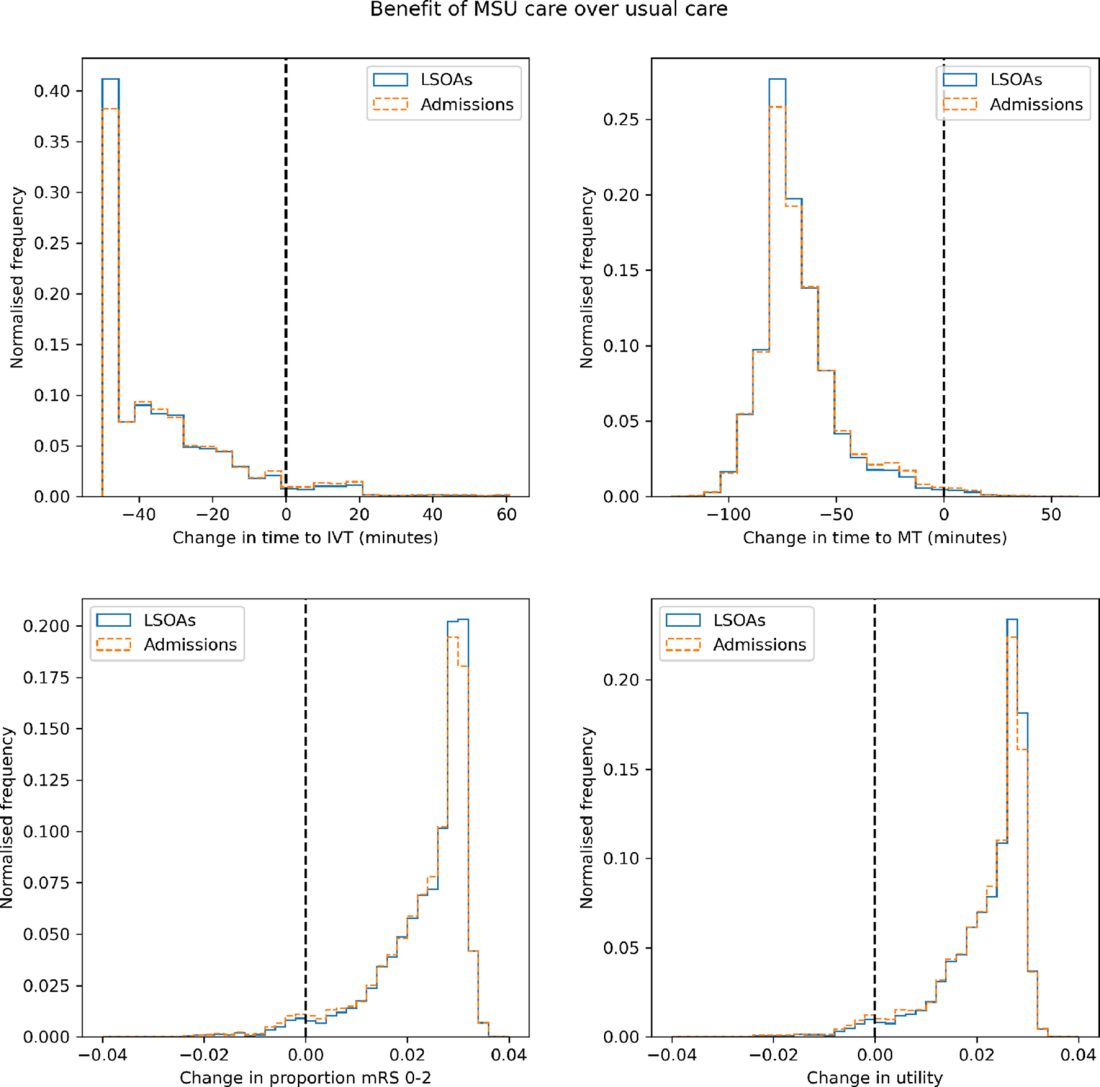
Distribution of benefit of MSU care over usual care across LSOAs (assuming 70% nLVO, 30% LVO in the treated population, with LVO receiving IVT/MT in combination). Benefit is described either as even across LSOAs (solid line), or weighted by admissions by LSOA (dotted line). Histograms show change in time to IVT (top left), time to MT (top right), proportion mRS 0–2 post stroke (bottom left), or utility (bottom right)

Figure 8 shows histograms of benefit of MSU care over usual care for the modelled population, with benefit represented as time to treatment, proportion of patients with outcome mRS 0–2 after stroke, and utility. For most LSOAs MSU care improves the time to IVT and MT, but some areas have worsened times. This is reflected in most areas having a benefit of about a 0.03 increase in the proportion of patients with mRS 0–2 after stroke, and similarly a benefit of about a 0.03 increase in utility after stroke. Some areas though have reduced benefit, or even disbenefit of using MSUs. The maximum benefit is about 0.04 improvement in both the proportion of patients with mRS 0–2 after stroke and utility after stroke.

### Varying number of MSU base locations

**Fig. 9:**
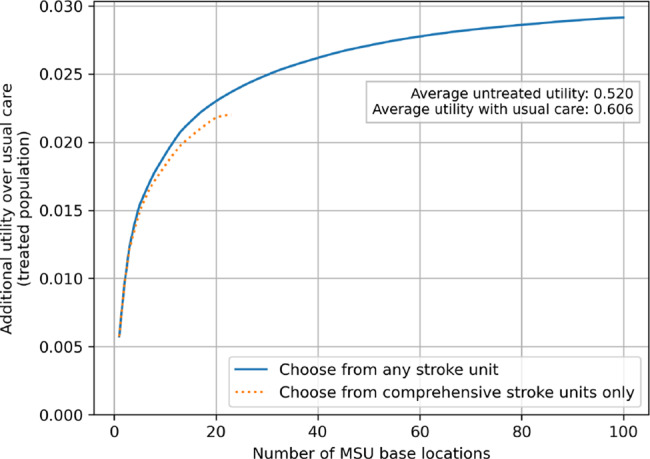
Increasing number of MSU base locations, with selection by a greedy algorithm based on improvements in utility. The additional utility is for those patients treated by MSU rather than usual care. Units were chosen either from any acute stroke centre type (solid line) or only from CSCs (dashed line). Utility for the untreated population was 0.520, which was increased to 0.602 with usual care

A greedy algorithm was used to select MSU base locations in which MSU base locations are sequentially added one at a time, with each new location selected based on the best possible improvement in utility by adding one more unit. The utility gain is calculated for those patients treated by an MSU rather than with usual care. As the number of MSU base locations increased, the benefit of MSU care over usual care increased (Fig. 9), but with diminishing returns. The advantage of MSU care over usual care improved utility by 0.020, 0.024, 0.027, and 0.29 with 10, 25, 50 and 100 MSU base locations when MSU base locations are chosen from any stroke unit type. There were 3 CSCs in the first 10 selections, and 8 CSCs in the first 20 selections. With MSU base locations at the 23 current CSCs, the net utility benefit over usual care was 0.022. With the same number of MSU base locations being selected from any stroke centre type, the utility benefit can be increased to 0.023.

### Proportion of patients receiving reperfusion

In our modelling we have focussed on the benefit for those who receive IVT or MT. Overall, the central probability across realistic scenarios is one extra independent-living outcome for every 50 patients treated. IVT and MT rates vary significantly between countries [32], but we might take a realistic target of 20% of patients receiving IVT or MT, in which case there would be one extra independent-living outcome for every 250 confirmed stroke patients (ischaemic and haemorrhagic) where the MSU is dispatched. Not all patients suspected of having stroke at ambulance dispatch will have a confirmed stroke; positive predicted value of suspected stroke at emergency dispatch is about 50% [32], in which case there would be one extra independent-living outcome for every 500 of all patients to whom the MSU is dispatched.

## Discussion

MSUs have been shown to have clear benefit in metropolitan areas [8, 9]. Our focus has been on predicting the benefit (or disbenefit) of MSUs across a wider geography, where MSUs may have poorer response times, but may still benefit particular patients by faster direct access to an MT-capable centre. To understand these complex geographic effects we have chosen to use modelling, based on predicting outcomes depending on times to both IVT and MT. The modelling of clinical effect was performed by synthesising known relationships between treatment times and outcomes for IVT and MT, while also extending the predictions to the expected mix of patients in real-world settings. By predicting the full range of mRS scores we can also estimate utility scores with different treatment options.

In this first study to model implementation of MSUs across a whole health system, overall we found a relatively marginal predicted benefit from the widespread adoption of MSUs across the whole of England (58 million population). Using plausible process timings we found the magnitude of benefit was likely to be an increase of 0.015 to 0.030 in health utility or 0.015 to 0.030 in the proportion of independent patients at 6 months after their stroke event. The benefit to patients with nLVO was around 0.015 in the two measures. The benefit to patients with LVO was larger (around 0.035 in the two measures), with the majority of this benefit coming from the direct conveyance of patients with LVO to an MT-capable centre, avoiding the need for inter-hospital transfers and their associated delays, though it is possible that those delays may be reduced by optimisation of other aspects of the current pathway. The benefit from earlier IVT is similar to that modelled by Holodinsky et al. [17]. Benefit was larger in the regions within reasonable travel distances of the MSU base locations, which would have similar catchments to those in the clinical trials of MSUs.

We have directly modelled only the ischaemic stroke population treated with IVT or MT. The overall net benefit will be diluted by patients the MSU is dispatched to who are either stroke patients who do not receive or are excluded from IVT or MT (including those with haemorrhage), or who are not confirmed to have had a stroke. In the treated population, across all of England, for every 100 patients suitable for IVT or MT, there will likely be 1–3 more people who can live independently because of earlier treatment. If only about 1 in 5 stroke patients are suitable candidates for IVT or MT, the MSU would need to attend approximately 250 stroke patients for every one extra independent-living outcome. If about half of the patients whom the MSU is dispatched to are actual strokes (the others being stroke mimics), the MSU would need to attend approximately 500 patients for every one extra independent-living outcome.

We found that the benefit of MSU care over usual care was critically dependent on rapid dispatch of the MSU and relatively rapid IVT (30 minutes) on-scene. MSUs are therefore not an alternative to the optimisation of day-to-day operational activities in ambulances or in hospitals. Additionally, if current processes are improved, the benefit of MSUs will be diminished. For example, ambulance on-scene times have been reported to have a median value of 33 min in a UK setting [33], compared to 15 min in a US setting [34]. Therefore, there may be potential to reduce onset-to-treatment times in usual care by at least 15 min by optimising usual pre-hospital care.

The benefit of MSU care diminished with distance from the MSU base location, though there was a halo effect for patients with LVO, with patients benefiting from direct transfer to MT-capable centres without excessive MSU arrival times. The diminishing benefit of MSUs is similar to that observed in clinical trials, where in Berlin the advantage of MSU care over usual care, when considering time to treatment, fell with distance from the MSU base location [35]. MSUs centred in metropolitan areas are therefore not a solution to significantly improving stroke outcomes in more remote locations. However if MSUs were based in remote locations there would be a challenge of long MSU travel times if the MSU is to maximise the number of patients seen each day. Additionally, MSUs will only partially solve the challenge of timely access to MT from remote areas. Maximising the net benefit of MSUs may therefore be at the cost of worsening equity of access to emergency stroke reperfusion therapies, as when MSUs are placed in metropolitan areas they improve access to care for those that already have the best access. Therefore care providers should also consider combining MSUs with other approaches which could reduce inequity such as CSC configurations and selection of patients for direct admission during standard ambulance assessment.

We modelled net delays in MT of 15–90 min added to the inter-hospital travel time. In a study of a stroke registry Froehler *et al.* found that, on average, MT was delayed by 75 min plus travel time [36]. Our exploration of possible delay times is biased more towards these processes improving as MT becomes more established, but in some particular instances transfer-related delays may be longer, which would improve the benefit of using MSU for patients that are suitable candidates for MT.

As the benefit of CSCs varies with patient location, a possible strategy of deployment of MSUs is to deploy them selectively for areas of maximum benefit, rather than planning to cover all of England.

We have compared MSU care to usual care, which will involve inter-hospital transfer for those patients first attending an IVT-only centre. We saw that a significant part of the benefit from MSU care for patients with LVO came from avoiding such transfers, with the patient being taken directly to an MT-capable centre. Such benefit may be achieved in other ways, such as use of clinical symptom scoring for pre-hospital section of patients likely to benefit from MT, or by achieving large reductions in door-in-door-out times at PSCs [37].

We have modelled MSUs being based at stroke centres. We compared basing MSUs at just CSCs (offering both IVT and MT) or at any type of acute stroke centre. The difference between these two approaches was marginal - likely because CSCs tend to be sited within large dense population centres, which gives them rapid access to large numbers of people. It is possible to site MSUs in other locations, such as ambulance stations, though most benefit will accrue from locating them in or near population centres, which is where stroke centres generally exist. As the number of MSU base locations is increased, the possible benefit increases, but with diminishing returns.

A possible difference between our model and real-world use of MSUs is that we assume a similar propensity for clinicians to give IVT and MT between both MSU care and usual care for patients within the time window for treatment. This assumption allows us to isolate geographic effects. However, we know different stroke clinicians and teams vary in their propensity to use IVT [38, 39], and there are other organisational barriers to use of IVT [40]. It is likely that MSUs will be staffed by clinicians more confident in using IVT, and organisational barriers to IVT delivery may be overcome, and so IVT use may increase not from the altered times to IVT, but by the patient being seen by clinicians more confident in using IVT and in a setting that has eliminated organisational barriers to IVT. This could partly explain why Chen et al. [9] found such a significant increase (34%) in IVT use in MSU trials; it seems unlikely that this degree of change could come just from the modest speed improvements offered by MSU. Another possible reason for higher IVT use in MSUs is that IVT may be ruled out where there are improving stroke symptoms [41], and this probably be less likely to be detected with rapid MSU-based assessment and treatment.

We present results for patients seen by the MSU compared with usual care. A challenge will be identification of the correct patients to dispatch the MSU. In a 2024 review of Emergency Medical Services dispatcher recognition of stroke [42], Wenstrup et al. found sensitivity varied from 17.9% to 83.0%. Sensitivity median and interquartile range was 56% (48%-63%). Positive predictive value (PPV) was reported in 12 papers and ranged from 24.0% to 87.7% with a median and interquartile range of 46% (42%-50%). Typically, therefore, half of stroke patients are not identified as such at ambulance dispatch, and only half of suspected stroke patients at dispatch are later confirmed to have a stroke. In one study it was found sensitivity for identifying stroke could be improved, but at the cost of PPV; in a study on the effect of training call handlers [43], on 464 patients, sensitivity improved from 63% to 80%, but PPV fell from 60.5% to 39.0%. Such uncertainty in the sensitivity and PPV of identification of stroke patients makes it difficult to predict how many stroke patients will be seen by MSUs, as that number is limited by both sensitivity of dispatch (where stroke patients are missed) but also by PPV which consumes MSU capacity, risking the MSU not being available as it attends a large number of non-stroke patients. In addition to concerns around identifying patients for MSU dispatch, other implementation concerns have been raised in a qualitative study of clinician views of MSUs [44]. This includes concerns over how they would be staffed, where they would be based, and whether they will increase or reduce equity of access to emergency stroke care.

Our study adds to the evidence base on how MSUs may affect times to MT, especially when used more widely than areas close to CSCs. When the catchment area of MSUs extends out beyond the usual catchment area of CSCs, the MSU captures patients who would otherwise go to a local IVT-only stroke centre and require onward transfer for MT - effectively functioning as an ambulance redirection intervention. MSUs therefore have potential to improve outcomes for those patients. This effect will be dependent on the MSU using CT-A to identify LVO patients and having reliable image interpretation immediately available. While CT-A has not been routinely used in all MSU models of care, it is increasingly being adopted to improve LVO diagnosis and also to improve MT workflows at the receiving hospital [16]. CT-A is likely to be an essential component of MSUs to maximise benefit to patients.

Estimates of the proportion of stroke patients who have LVO vary. A review by Rennert *et al.* identified estimates of 24% to 46% [45]. We have used an estimate of 30% to reflect the likely mix in the reperfusion treated population, but we have separated out benefit for nLVO and LVO so that our results may be interpreted for other mixes.

### Study limitations

Two key limitations have been discussed. Firstly, we isolated the geographic effects of MSUs and do not model how having an expert specialist team in a well-equipped MSU may increase IVT use simply by being more experienced, and so more confident, in use of IVT. Secondly, due to large uncertainties around sensitivity and PPV of identification of stroke patients for MSU dispatch, we limit our study to modelling of outcomes of those who are seen by the MSU. Real-world benefit will be diluted by stroke patients being missed, or by MSU capacity not being available when required, especially if capacity is constrained by low PPV of suspected stroke at dispatch. Similarly, for the same reason, we do not model how MSUs may affect emergency stroke admission numbers at hospitals (e.g. by changing effective catchment areas). It is possible that widespread use of MSUs could compromise the ability of some smaller hospitals to still provide IVT themselves due to loss of clinicians or experience. We also do not model other potential benefits of MSUs. For example, in haemorrhagic stroke there is potential to start reducing blood pressure sooner where that would benefit patients [46]. In non-stroke patients it is possible that improved diagnosis by the MSU could help identify the best destination for that patient sooner, or may give more confidence in leaving the patient at home, saving healthcare resources.

We have not modelled selection of late-presenting patients, or patients with unknown stroke onset time. These patients may be selected for IVT or MT based on advanced imaging. These patients would not be expected to follow the decline in effectiveness of IVT or MT described in analysis of how time-to-treatment affects outcomes [3, 4]. Further clinical trial data is needed to model how MSUs are likely to affect treatment of late-presenting patients or patients with unknown stroke onset time.

We have modelled MSUs compared with usual care. Alternative proposals for improving pre-hospital stroke care have also been suggested, that we have not sought to model here. For example, alternative approaches to ambulance redirection are available through use of clinical scale assessment triage [47], telemedicine [48], or near patient testing [49]. These alternative methods may offer advantages for patients further away from an MSU base. It is possible, therefore, that the best overall system could be a hybrid of MSU care and alternative methods of care where access to MSU care is likely to be limited.

## Conclusions

Overall, within our study assumptions, we found a relatively small benefit from MSUs across all of England, even with unrestricted MSU capacity. Benefit depends on efficiency of dispatch and treatment, and varies with geography. It is possible that more selective targeting of MSUs could help maximise benefit. A significant part of their potential benefit is derived from avoiding transfers for patients suitable for MT, reducing time to MT significantly. Maximising benefit from MSUs is critically dependent on rapid dispatch and fast on-scene IVT. However, there are other considerations such as resource limitations and implementation challenges, and MSUs should not be seen as an alternative to optimising day-to-day emergency stroke systems.

## Electronic supplementary material

Below is the link to the electronic supplementary material.

Supplementary Material 1

## Data Availability

General model code is available at https://github.com/stroke-modelling/muster2. Estimated travel times based on patient and hospital locations is available at https://gitlab.com/michaelallen1966/1811_lsoa_to_acute_hospital_travel/. Code for estimation of stroke outcome depending on time to IVT or MT is available at https://github.com/samuel-book/stroke_outcome/, and is available as a Python package at https://pypi.org/project/stroke-outcome/.

## References

[1] Feigin VL, Stark BA, Johnson CO, Roth GA, Bisignano C, Abady GG, Abbasifard M, Abbasi-Kangevari M, Abd-Allah F, Abedi V, Abualhasan A, Abu-Rmeileh NM, et al. Global, regional, and national burden of stroke and its risk factors, 1990–2019: a systematic analysis for the Global Burden of Disease Study 2019. Lancet Neurol. 2021;20:795–820. 34487721 10.1016/S1474-4422(21)00252-0 PMC8443449

[2] Luengo-Fernandez R, Violato M, Candió R, Leal J. Economic burden of stroke across Europe: a population-based cost analysis. Eur Stroke J. 2020;5:17–25. 32232166 10.1177/2396987319883160 PMC7092742

[3] Emberson J, Lees KR, Lyden P, Blackwell L, Albers G, Bluhmki E, Brott T, Cohen G, Davis S, Donnan G, Grotta J, Howard G, et al. Effect of treatment delay, age, and stroke severity on the effects of intravenous thrombolysis with alteplase for acute ischaemic stroke: a meta-analysis of individual patient data from randomised trials. Lancet. 2014;384:1929–35. 25106063 10.1016/S0140-6736(14)60584-5 PMC4441266

[4] Fransen PSS, Berkhemer OA, Lingsma HF, Beumer D, Berg LAVD, Yoo AJ, Schonewille WJ, Vos JA, Nederkoorn PJ, Wermer MJH, Walderveen MAAV, Staals J, et al. Time to Reperfusion and Treatment Effect for Acute Ischemic Stroke: a Randomized Clinical Trial. JAMA Neurol. 2016;73:190–96. 26716735 10.1001/jamaneurol.2015.3886

[5] Fassbender K, Walter S, Liu Y, Muehlhauser F, Ragoschke A, Kuehl S, Mielke O. “Mobile Stroke Unit” for Hyperacute Stroke Treatment. Stroke. 2003;34:e44–e44. 12750527 10.1161/01.STR.0000075573.22885.3B

[6] Taqui A, Cerejo R, Itrat A, Briggs FB, Reimer AR, Winners S, Organek N, Buletko AB, Sheikhi L, Cho S-M, Buttrick M, Donohue MM, et al. Reduction in time to treatment in prehospital telemedicine evaluation and thrombolysis. Neurology. 2017;88:1305–12. 28275084 10.1212/WNL.0000000000003786

[7] Walter S, Kostopoulos P, Haass A, Keller L, Lesmeister M, Schlechtriemen T, Roth C, Papanagiotou R, Grunwald L, Schumacher H, Helwig S, Viera J, et al. Diagnosis and treatment of patients with stroke in a mobile stroke unit versus in hospital: a randomised controlled trial. Lancet Neurol. 2012;11:397–404. 22497929 10.1016/S1474-4422(12)70057-1

[8] Fatima N, Saqqur M, Hussain MS, Shuaib A. Mobile stroke unit versus standard medical care in the management of patients with acute stroke: a systematic review and meta-analysis. Int J Stroke. 2020;15:595–608. 32515695 10.1177/1747493020929964

[9] Chen J, Lin X, Cai Y, Huang R, Yang S, Zhang G. A Systematic Review of Mobile Stroke Unit Among Acute Stroke Patients: time Metrics, Adverse Events, Functional Result and Cost-Effectiveness. Front Neurol. 2022;13:803162. 35356455 10.3389/fneur.2022.803162 PMC8959845

[10] Turc G, Hadziahmetovic M, Walter S, Churilov L, Larsen K, Grotta JC, Yamal J-M, Bowry R, Katsanos AH, Zhao H, Donnan G, Davis SM, et al. Comparison of Mobile Stroke Unit With Usual Care for Acute Ischemic Stroke Management: a Systematic Review and Meta-analysis. JAMA Neurol 2022. 10.1001/jamaneurol.2021.5321 PMC8822443 35129584

[11] Navi BB, Audebert HJ, Alexandrov AW, Cadilhac DA, Grotta JC. PRESTO (Prehospital Stroke Treatment Organization) Writing GrouP Mobile Stroke Units: evidence, Gaps, and Next Steps. Stroke 2022;53:2103–13. 35331008 10.1161/STROKEAHA.121.037376

[12] Ebinger M, Siegerink B, Kunz A, Wendt M, Weber JE, Schwabauer E, Geisler F, Freitag E, Lange J, Behrens J, Erdur H, Ganeshan R, et al. Association Between Dispatch of Mobile Stroke Units and Functional Outcomes Among Patients With Acute Ischemic Stroke in Berlin. JAMA. 2021;325:454–66. 33528537 10.1001/jama.2020.26345 PMC7856548

[13] Grotta JC, Yamal J-M, Parker SA, Rajan SS, Gonzales NR, Jones WJ, Alexandrov AW, Navi BB, Nour M, Spokoyny I, Mackey J, Persse D, et al. Prospective, Multicenter, Controlled Trial of Mobile Stroke Units. N Engl J Med. 2021;385:971–81. 34496173 10.1056/NEJMoa2103879

[14] Czap AL, Alexandrov AW, Nour M, Yamal J-M, Wang M, Jacob AP, Parker SA, Tariq MB, Rajan SS, Alexandrov AV, Jones WJ, Navi BB, et al. Impact of Mobile Stroke Units on Patients With Large Vessel Occlusion Acute Ischemic Stroke: a Prespecified BEST-MSU Substudy. Stroke Vasc Interv Neurol. 2024;4:e001095. 10.1161/SVIN.123.001095 PMC12778464 41586049

[15] Helwig SA, Ragoschke-Schumm A, Schwindling L, Kettner M, Roumia S, Kulikovski J, Keller I, Manitz M, Martens D, Grim D, Walter S, Lesmeister M, et al. Prehospital Stroke Management Optimized by Use of Clinical Scoring vs Mobile Stroke Unit for Triage of Patients With Stroke: a Randomized Clinical Trial. JAMA Neurol. 2019. 10.1001/jamaneurol.2019.2829 PMC6724153 31479116

[16] Czap AL, Singh N, Bowry R, Jagolino-Cole A, Parker SA, Phan K, Wang M, Sheth SA, Rajan SS, Yamal J-M, Grotta JC. Mobile Stroke Unit Computed Tomography Angiography Substantially Shortens Door-to-Puncture Time. Stroke. 2020;51:1613–15. 32295510 10.1161/STROKEAHA.119.028626 PMC7188560

[17] Holodinsky Jessalyn K, Noreen K, Charlotte Z, Ospel Johanna M, Luke Z, Wilson Alexis T, Hill Michael D, Mayank G. In What Scenarios Does a Mobile Stroke Unit Predict Better Patient Outcomes? Stroke. 2020;51:1805–12. 32389068 10.1161/STROKEAHA.119.028474

[18] Office of National Statistics. https://WWW.ons.gov.uk/methodology/geography/ukgeographies/statisticalgeographies

[19] Lees KR, Bluhmki E, von Kummer R, Brott TG, Toni D, Grotta JC, Albers GW, Kaste M, Marier JR, Hamilton SA, Tilley BC, Davis SM, et al. Time to treatment with intravenous alteplase and outcome in stroke: an updated pooled analysis of ECASS, ATLANTIS, NINDS, and EPITHET trials. Lancet. 2010;375:1695–703. 20472172 10.1016/S0140-6736(10)60491-6

[20] Goyal M, Menon BK, Van Zwam WH, Dippel DWJ, Mitchell PJ, Demchuk AM, Dávalos A, Majoie CBLM, Van Der Lugt A, De Miquel MA, Donnan GA, Roos YBWEM, et al. Endovascular thrombectomy after large-vessel ischaemic stroke: a meta-analysis of individual patient data from five randomised trials. Lancet. 2016;387:1723–31. 26898852 10.1016/S0140-6736(16)00163-X

[21] Duvekot MHC, Venema E, Rozeman AD, Moudrous W, Vermeij FH, Biekart M, Lingsma HF, Maasland L, Wijnhoud AD, Mulder LJMM, Alblas KCL, van Eijkelenburg RPJ, et al. Comparison of eight prehospital stroke scales to detect intracranial large-vessel occlusion in suspected stroke (PRESTO): a prospective observational study. Lancet Neurol. 2021;20:213–21. 33422191 10.1016/S1474-4422(20)30439-7

[22] De la Ossa Herrero N, Carrera D, Gorchs M, Querol M, Millan M, Gomis M, Dorado L, López Cancio E, Hernández-Pérez M, Chicharro V, Escalada X, Jiménez X, et al. Design and Validation of a Prehospital Stroke Scale to Predict Large Arterial Occlusion The Rapid Arterial Occlusion Evaluation Scale. Stroke. 2013;45. 10.1161/STROKEAHA.113.003071 24281224

[23] GitHub. https://github.com/stroke-modelling/muster2

[24] Moseley L, McMeekin P, Allen M, Ford GA, James M, Laws A, McCarthy S, McClelland G, Park LJ, Pearn K, Phillips D, Price C, et al. Co-design of a Mobile Stroke Unit pathway highlights uncertainties and trade-offs for viable system-wide implementation in the English and Welsh NHS. Res Sq Preprint. 2024.https://www.researchsquare.com/article/rs-5409053/vl. 10.1186/s12873-025-01243-7 PMC12147245 40484953

[25] GitLab. https://gitlab.com/michaelallenl966/1811_lsoa_to_acute_hospital_travel/

[26] GitHub. https://github.com/Samuel-booh/stroke_outcome/

[27] GitHub. https://samuel-book.github.io/stroke_outcome/intro,html

[28] PyPI. https://pypi.org/project/stroke-outcome/

[29] Quinn TJ, Dawson J, Walters MR, Lees KR. Functional Outcome Measures in Contemporary Stroke Trials. Int J Stroke. 2009;4:200–05. 19659822 10.1111/j.1747-4949.2009.00271.x

[30] Wang X, Moullaali TJ, Li Q, Berge E, Robinson TG, Lindley R, Zheng D, Delcourt C, Arima LL, Song L, Chen X, Yang J, et al. Utility-Weighted Modified Rankin Scale Scores for the Assessment of Stroke Outcome. Stroke. 2020;51:2411–17. 32640944 10.1161/STROKEAHA.119.028523

[31] Hassan AE, Zaidat OO, Nanda A, Atchie B, Woodward K, Doerfler A, Tomasello A, Fifi JT. Impact of interhospital transfer vs. direct admission on acute ischemic stroke patients: a subset analysis of the COMPLETE registry. Front Neurol. 2022;13. 10.3389/fneur.2022.896165 PMC9397115 36016541

[32] Global stroke statistics 2023: availability of reperfusion services around the world. 19. ISSN. 2024:1747–4930. 10.1177/17474930231210448 PMC10903148 37853529

[33] McClelland G, Burrow E, Alton A, Shaw L, Finch T, Price C. What factors contribute towards ambulance on-scene times for suspected stroke patients? An observational study. Eur Stroke J. 2023;8:492–500. 37231700 10.1177/23969873231163290 PMC10334177

[34] Patel MD, Brice JH, Moss C, Suchindran CM, Evenson KR, Rose KM, Rosamond WD. An Evaluation of Emergency Medical Services Stroke Protocols and Scene Times. Prehosp Emerg Care. 2014;18:15–21. 24028711 10.3109/10903127.2013.825354 PMC3973028

[35] Koch PM, Kunz A, Ebinger M, Geisler F, Rozanski M, Waldschmidt C, Weber JE, Wendt M, Winter B, Zieschang K, Bollweg K, Kaczmarek S, et al. Influence of Distance to Scene on Time to Thrombolysis in a Specialized Stroke Ambulance. Stroke. 2016;47:2136–40. 27328702 10.1161/STROKEAHA.116.013057

[36] Froehler MT, Saver JL, Zaidat OO, Jahan R, Aziz-Sultan MA, Klucznick RP, Haussen DC, Hellinger FR, Yavagal DR, Yao TL, Liebeskind DS, Jadhav AP, et al. Interhospital Transfer Prior to Thrombectomy is Associated with Delayed Treatment and Worse Outcome in the STRATIS Registry. Circulation. 2017. 10.1161/CIRCULATIONAHA.117.028920 PMC5732640 28943516

[37] Pérez de la Ossa N, Abilleira S, Jovin TG, García-Tornei Á, Jimenez X, Urra X, Cardona R, Cocho D, Purroy F, Serena J, San Román Manzanera L, Vivanco-Hidalgo RM, et al. Effect of Direct Transportation to Thrombectomy-Capable Center vs Local Stroke Center on Neurological Outcomes in Patients With Suspected Large-Vessel Occlusion Stroke in Nonurban Areas: the RACECAT Randomized Clinical Trial. JAMA. 2022. 10.1001/jama.2022.4404 PMC9073661 35510397

[38] De Bnin A, Flynn D, Ternent L, Price CL, Rodgers H, Ford GA, Rudd M, Lancsar E, Simpson S, Teah J, Thomson RG. Factors that influence clinicians’ decisions to offer intravenous alteplase in acute ischemic stroke patients with uncertain treatment indication: results of a discrete choice experiment. Int J Stroke. 2018;13:74–82. 28134031 10.1177/1747493017690755

[39] Pearn K, Allen M, Laws A, Monks T, Everson R, James M. What would other emergency stroke teams do? Using explainable machine learning to understand variation in thrombolysis practice. Eur Stroke J. 2023;8. 10.1177/23969873231189040 PMC10683721 37480324

[40] Meurer WJ, Majersik JJ, Frederiksen SM, Kade AM, Sandretto AM, Scott PA. Provider perceptions of barriers to the emergency use of tPA for acute ischemic stroke: a qualitative study. BMC Emerg Med. 2011;11(5). 10.1186/1471-227X-11-5 PMC3112102 21548943

[41] Balucani C, Levine SR. Mild Stroke and Rapidly Improving Symptoms: it’s Not Always A Happy Ending. Stroke. 2011;42:3005–07. 21903958 10.1161/STROKEAHA.111.628701 PMC3410646

[42] Wenstrup J, Hestoy BH, Sagar MV, Blomberg SNF, Christensen H, Christensen HC, Kruuse C. Emergency Medical Services dispatcher recognition of stroke: a systematic review. Eur Stroke J. 2024;9:283–94. 38174575 10.1177/23969873231223339 PMC11318428

[43] Watkins CL, Leathley MJ, Jones SP, Ford GA, Quinn T, Sutton CJ. Training emergency services’ dispatchers to recognise stroke: an interrupted time-series analysis. BMC Health Serv Res. 2013;13:318. 23947656 10.1186/1472-6963-13-318 PMC3751943

[44] Moseley L, McMeekin P, Price C, Shaw L, Allen M, Ford GA, James M, Laws A, McCarthy S, McClelland G, Park LJ, Pearn K, et al. Practitioner, patient and public views on the acceptability of Mobile Stroke Units in England and Wales: a mixed methods study. medRxiv. 2024;10.1101/2024.08.26.24312612. 10.1371/journal.pone.0310071 PMC11753667 39841768

[45] Rennert RC, Wali AR, Steinberg JA, Santiago-Dieppa DR, Olson SE, Pannell JS, Khalessi AA. Epidemiology, Natural History, and Clinical Presentation of Large Vessel Ischemic Stroke. Neurosurgery. 2019;85(Suppl 1):S4–S8. 31197329 10.1093/neuros/nyz042 PMC6584910

[46] Li G, Lin Y, Yang J, Anderson CS, Chen C, Liu F, Billot L, Li Q, Chen X, Liu X, Ren X, Zhang C, et al. Intensive Ambulance-Delivered Blood-Pressure Reduction in Hyperacute Stroke. N Engl J Med. 2024;390:1862–72. 38752650 10.1056/NEJMoa2314741

[47] Dekker L, Daems JD, Ali M, Duvekot MH, Nguyen TMT, Venema E, Durieux MD, van Zwet EW, Moudrous W, van den Wijngaard IR, Kerkhoff H, Lingsma HF, et al. Prehospital Large-Vessel Occlusion Stroke Detection Scales. Neurology. 2025;104:e213570. 40198869 10.1212/WNL.0000000000213570 PMC11984832

[48] Sarpourian F, Marzaleh MA, Aghda SAF, Zare Z. Application of Telemedicine in the Ambulance for Stroke Patients: a Systematic Review. Prehosp Disaster Med. 2023;38:774–79. 37877359 10.1017/S1049023X23006519

[49] Shaw L, Burgess D, Dixit A, Gaude E, Lendrem C, McClelland G, White P, Williams C, Zhu G, Price C. Rapid Assay Diagnostic for Acute Stroke Recognition (RADAR): study protocol for a diagnostic accuracy study. BMJ Open. 2024;14:e087130. 39122395 10.1136/bmjopen-2024-087130 PMC11331886

