# Supplementary material for: Modelling the potential clinical benefit of mobile stroke units in England
Source: BMC Emerg Med. 2025 Jul 1;25:111. doi: 10.1186/s12873-025-01242-8 (PMC12219994; doi:10.1186/s12873-025-01242-8)
Supplement: Supplementary file 1 — Supplementary Material 1 [file 12873_2025_1242_MOESM1_ESM.pdf]

Modelling the potential clinical benefit of mobile stroke units in England

Anna Laws[1,2], Michael Allen[*1,2], Jason Scott[3], Lisa Moseley[3], Kerry Pearn[1,2], Gary Ford[4], Chris Price[5], Phil White[5], Graham McClelland[3,7], Lisa Shaw[5], Daniel Phillips[6], Dave Wilson[6], Peter McMeekin[3], and Martin James[1,2]

[1]University of Exeter Medical School, Exeter, UK, [2]NIHR South West Peninsula Applied Research Collaboration (ARC), [3]Northumbria University, Newcastle-upon-Tyne, UK, [4]University of Oxford, Oxford, UK, [5]University of Newcastle, Newcastle-upon-Tyne, UK, [6]East of England Ambulance Service NHS Trust, Royston, UK, [7]North East Ambulance Service NHS Foundation Trust, Newcastle-upon-Tyne, UK, [*]Corresponding author: m.allen@exeter.ac.uk

Keywords: Stroke, Mobile Stroke Unit, Thrombolysis, Thrombectomy

A Supplementary material - Outcome modelling

A.1 Modified Rankin scale

We used modified Rankin Scale (mRS) at 3–6 months as a measure of outcome. mRS is the instrument most commonly used to describe the functional outcome post-stroke [1], describing the independence of living on a scale of 0 (no disability) to 5 (severe disability requiring constant nursing attention), with death assigned an mRS of 6. A commonly used surrogate for independent living is mRS 0–2 at 3–6 months. The health utility values for each mRS level were taken from Wang *et al.* [2]. These were 0.97, 0.88, 0.74, 0.55, 0.20, -0.19 and 0.0 mRS 0-6. The mean mRS score, the mean utility, and the proportion of patients with mRS 0–2 in a given mRS distribution can be compared between scenarios. Table S1 shows a description of each mRS category.

Table S1: Description of modified Rankin Scale (mRS)categories

mRS	Description
0	No symptoms.
1	No significant disability. Able to carry out all usual activities, despite some symptoms.
2	Slight disability. Able to look after own affairs without assistance but unable to carry out all previous activities.
3	Moderate disability. Requires some help, but able to walk unassisted.
4	Moderately severe disability. Unable to attend to their own bodily needs without assistance and unable to walk unassisted.
5	Severe disability. Requires constant nursing care and attention, bedridden, incontinent.
6	Dead.

A.2 Treatment of ischaemic stroke

Reperfusion treatment aims to restore blood flow after an ischaemic stroke. There are two potential reperfusion treatments:

- *Thrombolysis* (also known as intravenous thrombolysis, IVT) is a medical therapy in which clot-busting drugs are used to reduce or remove the blood clot. It is potentially of use in both nLVO and LVO.
- *Thrombectomy* (also known as mechanical thrombectomy, MT) is the physical removal of a clot, by a mesh device under X-ray imaging guidance. Thrombectomy is suitable only for clots in a large vessel (these generally cause the worst strokes). It is potentially of use in LVO, especially those LVO with stroke severity of NIHSS 6+, which comprise 80% of LVO [3].

A.3 Outcome modelling overview

Detailed methods and code used for modelling these outcomes are available [4], with methods described as an online book [5]. The outcome model is available as a PyPI package for Python [6].

We used modified Rankin Scale (mRS) at 3–6 months as a measure of outcome in different scenarios. The mean mRS score, mean utility and proportion of patients with mRS 0–2 in a given mRS distribution were compared between scenarios.

We calculated the patients mRS outcome distribution based on time to treatment for three cohorts of treated patients: 1) Patients with nLVO treated with IVT; 2) Patients with LVO treated with IVT alone; 3) Patients with LVO treated with IVT and MT. For each patient-treatment cohort we calculated an mRS distribution for treatment at any given time by interpolating between the mRS distribution for treatment given at *t=0* (time of stroke onset) and the mRS distribution for treatment given at *t=No Effect* (time of no effect of treatment), assuming that log odds fall linearly over time, as used in clinical trial meta-analyses [7, 8].

The time to no effect was assumed to be 6.3 hours for IVT [7] and 8.0 hours for MT [8]. Our model did not include the selection of patients who may still benefit from treatment beyond these durations through the use of advanced imaging. This number is small, of late treatment is likely to be small for IVT, but is more substantial for MT – approximately 2,500 per year in England.

The model synthesised data from multiple sources (figure S1) including reperfusion treatment clinical trials and national stroke audit data for England and Wales. Predictions from the combined model were then compared against original clinical trials and other models. Details of how these data were synthesised are given in the sections below.

The model produces mRS probability distributions for nLVO treated with IVT and LVO treated with IVT and/or MT, with the benefit of treatment decreasing over time after the appearance of stoke (Figure S2).

A.4 Extrapolation of reperfusion effects back to time zero

In order to model the effectiveness of reperfusion at any given time point, we interpolated between the theoretical effectiveness at time zero (the time of stroke onset) and the time at which the treatment no longer has any

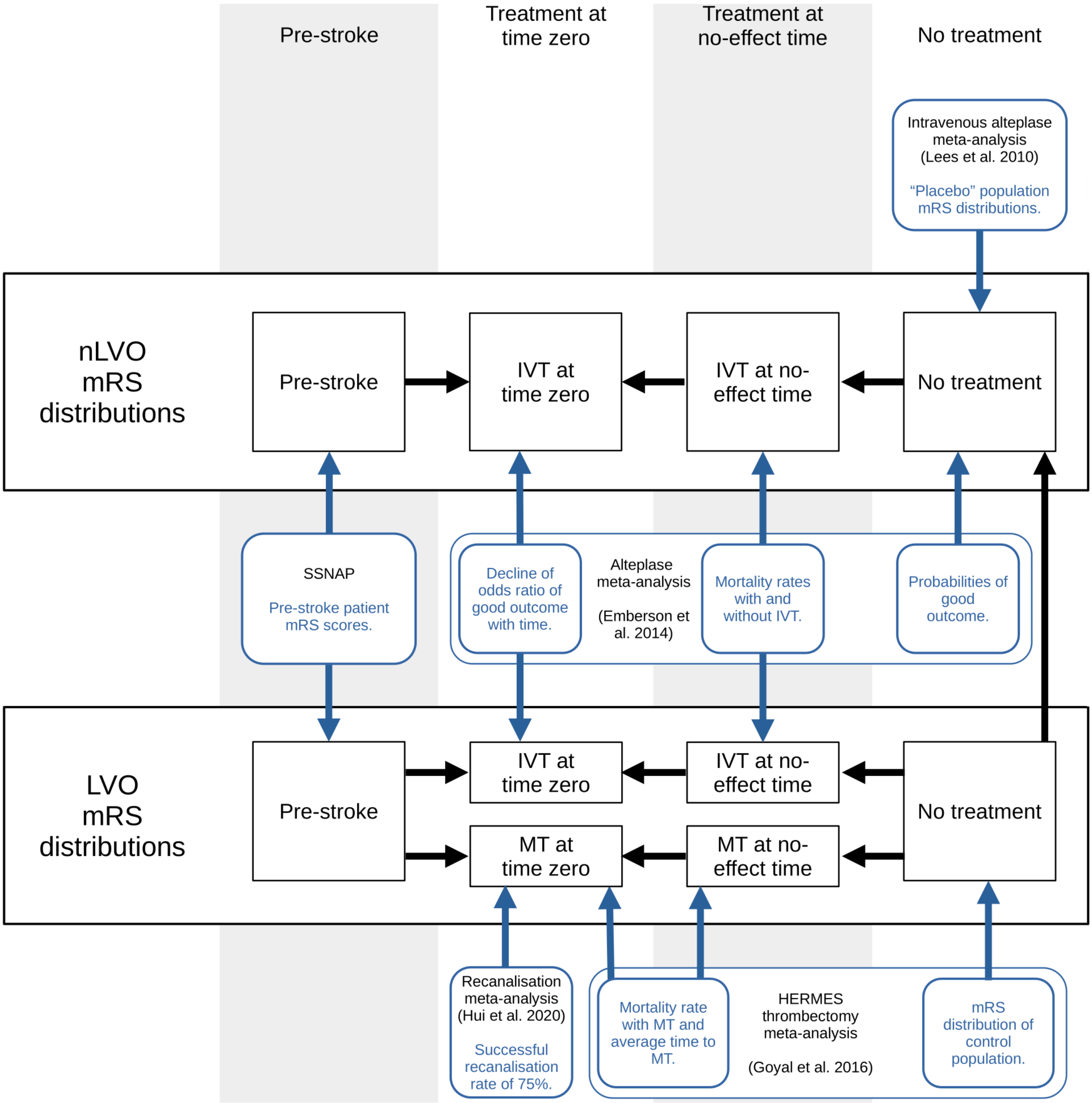

Figure S1: Summary of data sources used to estimate reference distributions.

clinical benefit (the time of no effect). Clinical trials model this decay as a linear decay in logarithmic odds of an improved outcome [7, 8].

A.5 Derivation of mRS distributions from reference data

A.5.1 General method

The steps used to create the mRS distributions were:

1. We estimated the mRS distributions of populations before their stroke.

   - These data were available in the UK national stroke audit (SSNAP) data.
   - We split the full cohort of patients into nLVO and LVO based on their NIHSS score (using NIHSS

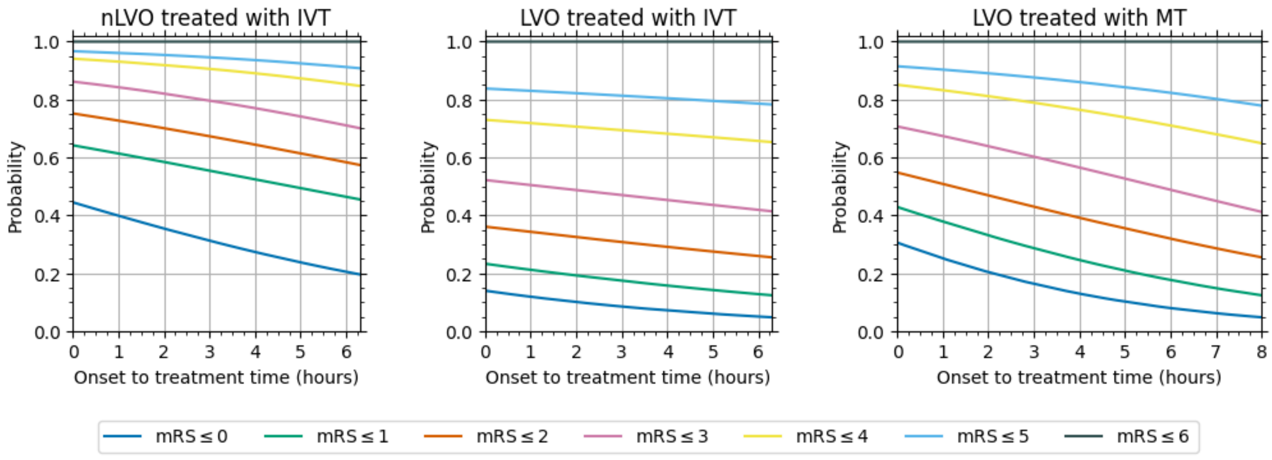

Figure S2: Model predictions of mRS distributions for nLVO and LVO depending on time to treatment with IVT (for nLVO or LVO) or MT (for IVT and LVO).

11+ as a surrogate indicator of a large vessel occlusion stroke that will be treatable by MT).

2. We estimated the mRS distributions of populations that received no treatment.
   - This data was available for two groups. The first group was patients with LVOs. The second group was a mix of patients with nLVOs and patients with LVOs.
   - We combined the groups and inferred the distribution for the population patients with nLVO (as nLVO-specific data is not available, but may be estimated by the diffence between LVO and mixed nLVO/LVO groups).
3. We estimated the mRS distributions of populations if they were treated at the time of no beneficial effect.
   - At the time of no effect, we assumed that patients given the treatment will see no benefit but are still exposed to a risk of death from treatment.
   - We take the distributions for populations that received no treatment and adjusted them for this predicted death rate in the absence of a beneficial treatment effect.
4. We estimated the mRS distributions of populations if they were treated at time zero (the time of stroke).
   - For IVT:
     – We took reference data points for mRS ≤ 1 at time zero and the mRS distributions at the time of no effect. From these data points, we were able to estimate the probability distribution at time zero.
     – Fixing this point, we take a weighted combination of the pre-stroke and no-effect distributions so that the combination's mRS $\leq$ point matches the fixed point.
   - For MT:
     – Define the time-zero distribution as a weighted combination of 75% of the pre-stroke distribution and 25% of the no-effect distribution.
     – Adjust the treatment-related death rate until the mRS distribution at a reference data time

Table S2: Pre-stroke mRS distributions for nLVO and LVO

mRS	0	1	2	3	4	5	6
nLVO	0.583	0.163	0.104	0.101	0.042	0.007	0
LVO	0.408	0.144	0.12	0.166	0.118	0.044	0

Table S3: No-treatment mRS distributions for nLVO and LVO

mRS	0	1	2	3	4	5	6
nLVO	0.198	0.262	0.120	0.128	0.148	0.062	0.082
LVO	0.050	0.079	0.136	0.164	0.247	0.135	0.189

matches the mortality rate at that reference time.

Figure S1 shows a summary of the data sources used to estimate the mRS distribution for each reference population.

A.5.2 Pre-stroke mRS distributions

The pre-stroke mRS distribution was from the national stroke audit for patients admitted to hospital with stroke between 2016 and 2018. A NIHSS of 0-10 was taken as a surrogate of LVO, and NIHSS 11+ as a surrogate of LVO. This cut-off was found to have maximum discrimination between LVO and nLVO [9]. Resulting pre-stroke mRS distributions are shown in Table S2.

A.5.3 No treatment mRS distributions

For LVO, the estimated post-stroke mRS distribution was taken from the control population of the HERMES trial [10]. Though the control group also included patients who had received thrombolysis, patients who had been successfully treated with thrombolysis would typically not have reached selection for trials on thrombectomy [11].

For nLVO, we take the control population from the thrombolysis clinical trials [7]. The meta-analysis provides the mRS breakdown for control and treated groups (which will contain a mix of nLVO and LVO). The analysis also provides the proportion of patients mRS 0-1 for control and treated groups broken down into NIHSS subgroups. Those in the NIHSS 0-10 groups are assumed to be nLVO. The proportion mRS 0-1 in those subgroups is used to infer the full separate mRS distribution for patients in NIHSS 0-10 groups.

Resulting pre-stroke mRS distributions are shown in Table S3.

A.5.4 Treatment at the no-effect time

For treatment at the no-effect time, we expect the distribution to be the same as untreated, but with some treatment-related deaths.

For IVT, the meta-analysis of clinical trials [7] reports deaths from intracranial haemorrhage. The IVT-related death rate is taken as the difference between treatment and control groups, split by NIHSS 0-10 (a surrogate for nLVO) and NIHSS 11+ (a surrogate for LVO).

For MT, treatment-related deaths must be inferred from available data. The steps were:

- We identified a reference data point where both the death rate and time to treatment were known. We use the mean time to treatment and the mortality rate from the HERMES meta-analysis [10].

- Use pre-stroke population as a surrogate for 'full recanalisation', but assume only 75% patients achieve recanalisation [12].
- Assume that the beneficial effect of thrombectomy decays to zero in 6 hours [8].
- Find the number of treatment-related deaths that would adjust the number of deaths at our given time point to the observed value. This figure is 4.0%.

Resulting estimates of treatment-related deaths are shown in table S4.

Table S4: Estimated treatment-related deaths

Patient group	Treatment-related death rate
nLVO with IVT	1.1%
LVO with IVT	3.4%
LVO with MT	4.0%

A.6 Interpolating effectiveness of treatment depending on time to treatment

Once mRS distributions have been calculated for treatment given at time of stroke onset (time zero) and time of no-effectiveness of treatment, we interpolate between the two distributions based on a linear change in log-odds of reaching any given mRS threshold (which is the relationship used in clinical trials of thrombolysis and thrombectomy [7, 8]). These may then be converted to odds, and then probability ($P = \frac{odds}{1+odds}$).

We assumed that the time of no-effect was 6.3 hours after stroke onset for IVT [7], and 8.0 hours for MT [8].

Figure S3 shows interpolation of the effectiveness of treatment, using LVO treated with MT as an example.

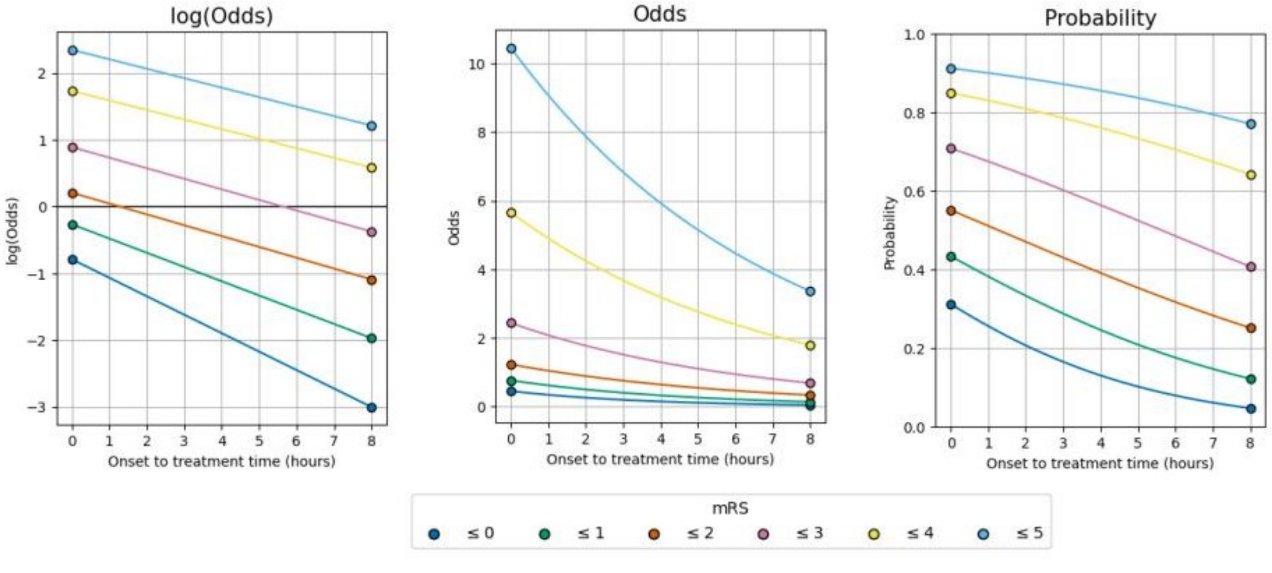

Figure S3: Predicting outcomes based on interpolation between time of stroke onset and time of no-effect of treatment. This examples shows the effect of MT for patients with LVO stroke.

A.7 Proportion of patients with large vessel occlusion

The proportion of patients with LVO depends on the population under study.

In the RACECAT trial of pre-hospital selection of patients with LVO [9], 34% of all stroke, and 41% of ischaemic stroke were LVO.

In a study analysing the proportion of stroke patients likely to benefit from thrombectomy McMeekin *et al.* [3] reported that 40% of ischaemic stroke patients had a LVO, with 80% of those (0r 32% of all ischaemic stroke) having NIHSS 6+ (suggesting they may benefit from thrombectomy).

In an analysis of SSNAP stroke audit data, for those arriving within 4 hours of stroke onset, 29% of all stroke and 34% of ischaemic stroke were NIHSS 11+ (a surrogate for LVO). Table S5 gives a more complete breakdown of SSNAP data.

Table S5: Proportion of patients by stroke subgroup and admission times (from SAMueL analysis*)

Admission type	All arrivals	Arrival within 6 hrs known onset	Arrival within 4 hrs known onset
Proportion all admissions	100	42.9	37.1
Proportion haemorrhagic	11.5	13.6	14.1
Proportion ischaemic	88.5	86.4	85.9
Proportion ischaemic with NIHSS 0-10	74.9	67.4	65.7
Proportion ischaemic with NIHSS 11+	25.1	32.6	34.3

*From `https://samuel-book.github.io/samuel-1/descriptive_stats/10_using_nihss_10_for_lvo.html`

In this study we assumed that the treatable population was compromised of 30% LVO (treatable with IVT and/or MT) and 70% nLVO (treatable with IVT only).

References for supplementary material

1. Quinn, T. J., Dawson, J., Walters, M. R. & Lees, K. R. Functional Outcome Measures in Contemporary Stroke Trials. *International Journal of Stroke* **4,** 200–205 (2009).
2. Wang, X., Moullaali, T. J., Li, Q., Berge, E., Robinson, T. G., Lindley, R., Zheng, D., Delcourt, C., Arima, H., Song, L., Chen, X., Yang, J., *et al.* Utility-Weighted Modified Rankin Scale Scores for the Assessment of Stroke Outcome. *Stroke* **51,** 2411–2417 (2020).
3. McMeekin, P., White, P., James, M. A., Price, C. I., Flynn, D. & Ford, G. A. Estimating the number of UK stroke patients eligible for endovascular thrombectomy. *European Stroke Journal* **2,** 319–326 (2017).
4. GitHub. *https://github.com/samuel-book/stroke_outcome/*
5. GitHub. *https://samuel-book.github.io/stroke_outcome/intro.html*
6. PyPI. *https://pypi.org/project/stroke-outcome/*
7. Emberson, J., Lees, K. R., Lyden, P., Blackwell, L., Albers, G., Bluhmki, E., Brott, T., Cohen, G., Davis, S., Donnan, G., Grotta, J., Howard, G., *et al.* Effect of treatment delay, age, and stroke severity on the effects of intravenous thrombolysis with alteplase for acute ischaemic stroke: A meta-analysis of individual patient data from randomised trials. *The Lancet* **384,** 1929–1935 (2014).
8. Fransen, P. S. S., Berkhemer, O. A., Lingsma, H. F., Beumer, D., Berg, L. A. v. d., Yoo, A. J., Schonewille, W. J., Vos, J. A., Nederkoorn, P. J., Wermer, M. J. H., Walderveen, M. A. A. v., Staals, J., *et al.* Time to Reperfusion and Treatment Effect for Acute Ischemic Stroke: A Randomized Clinical Trial. *JAMA Neurology* **73,** 190–196 (2016).

9. Pérez de la Ossa, N., Abilleira, S., Jovin, T. G., García-Tornel, Á., Jimenez, X., Urra, X., Cardona, P., Cocho, D., Purroy, F., Serena, J., San Román Manzanera, L., Vivanco-Hidalgo, R. M., *et al.* Effect of Direct Transportation to Thrombectomy-Capable Center vs Local Stroke Center on Neurological Outcomes in Patients With Suspected Large-Vessel Occlusion Stroke in Nonurban Areas: The RACECAT Randomized Clinical Trial. *JAMA* (2022).
10. Goyal, M., Menon, B. K., Van Zwam, W. H., Dippel, D. W. J., Mitchell, P. J., Demchuk, A. M., Dávalos, A., Majoie, C. B. L. M., Van Der Lugt, A., De Miquel, M. A., Donnan, G. A., Roos, Y. B. W. E. M., *et al.* Endovascular thrombectomy after large-vessel ischaemic stroke: A meta-analysis of individual patient data from five randomised trials. *The Lancet* **387,** 1723–1731 (2016).
11. Tsivgoulis, G., Katsanos, A. H., Schellinger, P. D., Köhrmann, M., Varelas, P., Magoufis, G., Paciaroni, M., Caso, V., Alexandrov, A. W., Gurol, E. & Alexandrov, A. V. Successful Reperfusion with Intravenous Thrombolysis Preceding Mechanical Thrombectomy in Large Vessel Occlusions. *Stroke* **49,** 232–235. (2025) (2018).
12. Hui, W., Wu, C., Zhao, W., Sun, H., Hao, J., Liang, H., Wang, X., Li, M., Jadhav, A. P., Han, Y. & Ji, X. Efficacy and Safety of Recanalization Therapy for Acute Ischemic Stroke With Large Vessel Occlusion. *Stroke* **51,** 2026–2035 (July 2020).
